# Droplet Electricity Generators With Maximized Energy Collection Zone Enabled by Aloe‐Inspired Midrib and Cuticle

**DOI:** 10.1002/adma.202523637

**Published:** 2026-03-12

**Authors:** Gibeom Lee, Eunbyeol Kim, Kyongtae Choi, Minjun Song, Sunmin Jang, Dongwhi Choi, Donghyun Seo, Min‐Gyu Lee, Younghoon Lee

**Affiliations:** ^1^ Department of Mechanical Engineering Kyung Hee University Yongin Republic of Korea; ^2^ Department of Mechanical Engineering Gachon University Seongnam Republic of Korea; ^3^ ES Advanced R&D Division LG Electronics Seoul Republic of Korea; ^4^ Semiconductor R&D Center Samsung Electronics Co., Ltd Yongin Republic of Korea

**Keywords:** droplet electricity generators, hydrogels, iontronics, triboelectric nanogenerators

## Abstract

Leveraging water as an abundant and continuously available natural resource, droplet electricity generators (DEGs) have gained significant attention for their ability to produce high instantaneous voltages from the simple impact of water droplets. However, conventional DEGs are fundamentally limited by their narrow energy collection zones, confined to regions immediately adjacent to the switch electrode. In nature, Aloe vera overcomes a similar spatial challenge by guiding scarce rainfall toward the root through its midrib‐like longitudinal ridge and water‐repellent wax‐rich cuticle—an integrated water‐guiding architecture that transports droplets over long distances with minimal loss. Here, inspired by this mechanism, we introduce a scalable DEG that maximizes the energy collection zone through an artificial droplet‐channeling strategy. A midrib‐inspired curvilinear geometry induces uni‐directional spreading upon impact, while an artificial hydrophobic cuticle composed of a hydrocarbon‐based OTS–squalane layer enables smooth, low‐retention sliding and robust interfacial stability. These features substantially expand the effective collection zone and yield approximately 236% higher charge output than a conventional DEG, unlocking new opportunities for large‐area, distributed, and environmentally adaptive droplet‐energy harvesting.

## Graphical abstract

An Aloe‐pinspired droplet electricity generator (A‐DEG) overcomes the limited energy collection zone of conventional DEGs by guiding impact droplets through a channeling midrib and artificial cuticle. The channeling midrib induces uni‐directional droplet spreading, while the artificial cuticle on the midrib further reinforces this behavior through its water repellency. It maximizes the energy collection zone and achieves approximately 236% higher charge output than conventional DEG.

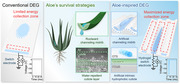

## 1 Introduction

With the increasing global emphasis on sustainable development, harvesting renewable energy from natural sources such as solar, wind, thermal gradients, and hydropower has become a critical area of research [1, 2, 3, 4, 5]. These energy sources offer eco‐friendly alternatives to fossil fuels and have found widespread use in various sectors [6]. Among them, as water covers over 70% of Earth's surface, its dynamic forms—including individual droplets—serve as abundant and continuously renewed mechanical energy sources throughout the hydrological cycle [7, 8]. Their transient mechanical energy, if effectively captured, can contribute to powering both compact electronics and distributed systems. Especially, harvesting energy from droplet motion across broader spatial domains opens new possibilities for integration into real‐world, large‐scale applications [9, 10]. As the demand for decentralized power generation and scalable, autonomous platforms continues to grow, droplet‐based energy harvesting presents a novel and adaptable solution within the broader landscape of renewable energy.

**FIGURE 1:**
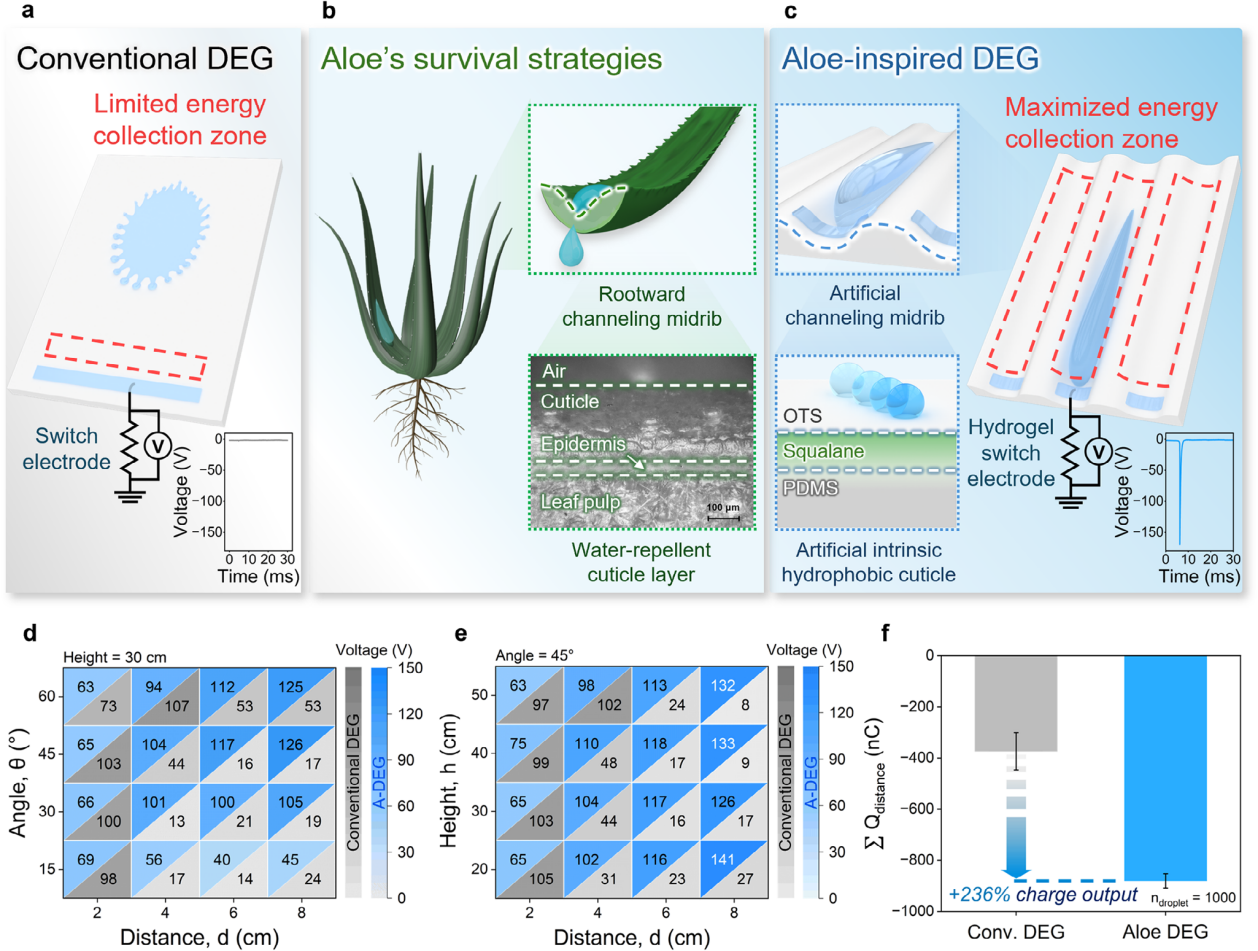
Overview of Aloe‐inspired droplet electricity generator (A‐DEG). (a) Limitation of conventional DEG. Energy harvesting of conventional DEG is restricted to droplet impacts only near the switch electrode. (b) Aloe's survival strategies in a harsh desert. Aloe has evolved a midrib‐like ridge structure for rootward water channeling, and a water‐repellent cuticle layer, enabling efficient collection and transport of water toward the root. (c) Conceptual diagram of A‐DEG. Energy collection zone is maximized by emulating Aloe's channeling midrib and hydrophobic cuticle, allowing the droplet to spread toward the switch electrode. Voltage output comparison between conventional DEG and A‐DEG with respect to distance, angles (d), and height (e). A‐DEG exhibits outstanding energy harvesting performance at distances of 4–8 cm. (f) Further enhanced distance utility of A‐DEG with a 236% increase in charge output.

Droplet‐mediated energy harvesting has evolved through multiple mechanisms—from contact electrification and evaporation‐induced ion gradient formation to capillary‐driven charge separation—enabling efficient conversion of transient droplet motion into electrical energy [11, 12, 13]. Among them, droplet electricity generators (DEGs) convert the mechanical energy of falling water droplets into electrical output through contact electrification and electrostatic induction, accompanied by ion redistribution arising from droplet deformation on the surface [14]. Despite their structural simplicity and their ability to generate high instantaneous voltages, most reported DEGs are based on nominally flat surfaces and therefore suffer from limited energy output arising from the small effective collection area. In conventional DEGs, energy harvesting is effective only when droplets impact near the electrodes, causing an inherently limited energy collection zone, as illustrated in Figure 1a. There have been several studies that attempted to expand the energy collection zone of conventional DEGs by increasing electrode density or introducing electrode arrays [15, 16]. As summarized in Table S1, however, they have not provided a fundamental solution for the inherent spatial limitation of DEGs, as the electrical output is mainly governed by the bulk effect [9, 10, 15, 16, 17, 18, 19]. The electrical output of a DEG is proportional to the spreading area of the droplet upon contact with the switch electrode. Therefore, excessive electrode density reduces the available space for the droplet spreading area, leading to paradoxically reduced voltage generation. For practical applications, expanding the effective energy collection region through controlled droplet guidance becomes essential, positioning the mitigation of spatial and efficiency constraints as a central direction for next‐generation DEG development.

To address this challenge, the survival strategies of Aloe vera in harsh desert environments offer an intriguing insight, as illustrated in Figure 1b. Aloe vera, a succulent plant, has evolved remarkable water‐guiding capabilities to survive in harsh environments with scarce rainfall. Along the longitudinal axis of its rosette‐arranged leaves, Aloe exhibits a ridge‐like, centrally curved structure that directs impinging water droplets toward the root zone [20, 21]. Although Aloe vera does not possess a botanical “midrib” in the strict anatomical sense, similar central ridge‐like leaf structures have been conventionally referred to as a midrib in prior Aloe‐related and biomimetic studies [22]. In this work, we adopt the term “midrib” in a functional sense to describe this longitudinal, rootward‐guiding structure responsible for uni‐directional water transport, rather than implying strict anatomical equivalence. Moreover, the hydrocarbon‐based hydrophobic cuticle layer further minimizes residual droplet retention, allowing droplets to slide smoothly along the channeling midrib and enhancing root‐directed water transport [23, 24]. The cuticle layer of aloe is known to function as a composite barrier, in which wax components are distributed both within the cutin scaffold and at the outermost surface, reducing droplet pinning while maintaining mechanical robustness [23, 25]. Inspired by this architecture, our artificial cuticle combines an OTS‐functionalized PDMS surface with a mobile hydrocarbon alkane oil that diffuses into the underlying PDMS matrix while remaining stabilized at the outermost surface, collectively enabling durable droplet mobility. Leveraging Aloe's water‐guiding mechanisms offers a promising strategy to overcome spatially constrained energy collection and enhance the overall performance of DEG.

Here, we introduce a scalable DEG that achieves a spatially maximized energy collection zone by guiding droplet pathways, inspired by Aloe's rootward water‐channeling strategy. A midrib‐inspired curvilinear geometry induces droplets to spread uni‐directionally upon impact, establishing a guided flow toward the single hydrogel switch electrode, as depicted in Figure 1c. This hydrogel‐based configuration ensures a mechanically stable and conformal interface with the soft polymer substrate, which effectively prevents interfacial delamination [19]. Moreover, the hydrogel inherently mitigates dehydration‐induced performance degradation by continuously absorbing moisture from the impinging droplets, thereby sustaining high electrical conductivity during prolonged operation [9]. An artificial hydrophobic cuticle formed from a hydrocarbon‐based OTS–squalane composite layer exhibits low droplet retention, enabling smooth sliding and continuous droplet transport with minimal residual liquid. The squalane infiltrated into the OTS‐functionalized surface layer improves mechanical robustness under consecutive droplet impact by suppressing crack growth through its strong affinity with OTS, ensuring a stable interfacial structure. It expands the effective energy collection zone and delivers approximately 275% higher charge output than a conventional DEG, highlighting the significant performance gains enabled by the Aloe‐inspired approach, which will be discussed in detail in the subsequent sections. This approach represents a step toward overcoming the long‐standing spatial constraints of DEGs and droplet‐based energy harvesting toward practical implementation.

## 2 Results and Discussion

### 2.1 Biomimetic Concepts and Electrical Characteristics of A‐DEG

Inspired by these biological strategies, an Aloe‐inspired droplet electricity generator (A‐DEG) integrates an artificial channeling midrib and an intrinsically hydrophobic artificial cuticle. The geometrically designed artificial midrib directs the uni‐directional spreading droplets toward the hydrogel switch electrode, thereby maximizing the energy collection zone regardless of impact position. However, PDMS‐based artificial midribs often suffer from unwanted droplet pinning due to intrinsic surface adhesion, which results in inefficient energy harvesting. Here, enhancing the inherent hydrophobicity of the PDMS surface is an effective approach. To date, PDMS surfaces have been functionalized using trichlorosilane‐based silanes such as (Heptadecafluoro‐1,1,2,2‐tetrahydrodecyl)trichlorosilane (HDFS) and octadecyltrichlorosilane (OTS) [26, 27]. The surface modification method is particularly effective because trichlorosilane‐based silanes readily form stable covalent linkages with siloxane‐based PDMS surfaces. However, the diffusion of low molecular weight oligomers from the PDMS bulk to the surface gradually deteriorates the performance by masking these nanometer‐scale trichlorosilane layers [28, 29]. Furthermore, slippery liquid‐infused porous surfaces (SLIPS) have been widely explored as an effective strategy to suppress droplet pinning by maintaining a lubricating liquid layer at the interface [30]. These approaches typically employ nanoscale surface texturing or porous architectures to physically retain the lubricant, and are well‐suited for many applications requiring robust liquid repellency. However, the incorporation of such texturing or porosity inevitably introduces additional fabrication complexity and processing steps. For practical applications, a surface modification strategy that can be directly applied to a nominally flat substrate without additional patterning is highly attractive. In parallel, fluorinated silane‐based surface modifications were intentionally not pursued in this study due to increasing concerns regarding environmental persistence and long‐term biocompatibility. To address the issue, an artificial cuticle was formed on the PDMS surface using OTS in combination with squalane, a hydrocarbon lubricant. This artificial cuticle significantly enhances droplet spreading and sliding along the rootward guiding midrib compared with bare PDMS.

To validate the effectiveness of the bio‐inspired design, the electrical outputs of the A‐DEG were compared with the pristine PDMS‐based conventional DEG. According to the triboelectric series, water acts as a tribo‐positive material, whereas both PDMS and squalane serve as tribo‐negative layers [31]. The distinct polarity difference facilitates effective contact electrification, thereby ensuring high voltage generation. The comparison was performed under key environmental parameters, including the impact distance between the droplet impact position and the hydrogel switch electrode (d), the tilting angle (θ), and the falling height (h), as indicated in Figure S1. Across a wide range of distances (2–8 cm) and tilting angles (15–60°), the A‐DEG consistently generates higher voltage outputs (40–126 V) than the conventional DEG (14–107 V), as shown in Figure 1d. An exception occurs at the closest distance (d = 2 cm), where the conventional DEG produces a higher voltage output (93.5 ± 13.8 V) compared to the A‐DEG (65.8 ± 2.5 V) due to the larger droplet spreading area by the bulk effect [17, 18]. Beyond this point, however, the performance of the conventional DEG rapidly decreases from 93.5 V at d = 2 cm to 28.3 V at d = 8 cm. In contrast, A‐DEG exhibits significantly increasing voltage output from 65.8 V at d = 2 cm to 100.3 V at d = 8 cm. At larger distances (d = 4–8 cm), the droplet on the conventional DEG is unable to maintain its spread while sliding across the surface. Instead, it shrinks before reaching the electrode, resulting in a reduced contact area and diminished voltage generation. Figure 1e reveals a similar tendency when varying the falling height from 20 to 50 cm. The A‐DEG consistently generates high voltage output of 65–141 V, whereas the conventional DEG generates lower voltage output of 8–105 V. The robustness across the height conditions highlights the strong practical applicability of the A‐DEG in real environments where droplet potential energy varies significantly. For an overall comparison across distances, the performance enhancement was quantified by integrating the current outputs of 1000 individual droplets to obtain the charge outputs, as presented in Figure 1f. Summing the integrated charge over the full distance range (2–8 cm) shows that the A‐DEG (−880 ± 28 nC) delivers approximately 236% more charge than the conventional DEG (−373 ± 73 nC). To further examine the statistical robustness and scalability of the device performance, the cumulative charge output was evaluated over a broad range of droplet impact counts, varying from 8 to 2000 as shown in Figure S2. Across this entire range, the enhancement ratio remained consistently high, varying only between 236% and 261%. These results indicate that the improved charge‐collection efficiency of the A‐DEG is not a short‐lived or stochastic phenomenon, but rather a stable and intrinsic performance characteristic that is preserved during prolonged operation.

### 2.2 Cuticle‐Inspired Surface Modification and Wetting Behavior

**FIGURE 2:**
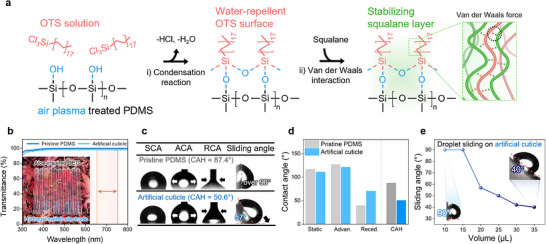
Analysis of water‐repellent capability of artificial cuticle inspired by the Aloe leaf cuticle. (a) Schematic illustration of the surface modification process for artificial cuticle. Trichloro(octadecyl)silane (OTS) forms covalent siloxane bonds with hydroxylated PDMS through a condensation reaction (i). Squalane (C_30_H_62_) interacts with C_18_ alkyl chains of OTS via van der Waals interactions (ii), thereby providing enhanced water repellency. (b) Transmittance spectra of artificial cuticle compared to pristine PDMS, which exhibits 97% transmittance at a wavelength of 550 nm. Artificial cuticle effectively transmits red light across 650–750 nm wavelength range. (c) Wettability comparison of pristine PDMS and artificial cuticle, characterized by static, advancing, receding contact angles, and sliding angles. (d) Reduced contact angle hysteresis (∆CAH = 36.8°) of the artificial cuticle indicates enhanced water droplet mobility. (e) Sliding behavior of water droplets on artificial cuticle. Sliding angle of water droplets was decreased (90–46°) with increasing volumes (10–35 µL) on artificial cuticle. Sliding angles of 20 µL water droplets on pristine PDMS (> 90°) and artificial cuticle (≈ 57°).

The Aloe leaf exhibits superior water repellency and a highly stable structure with a hydrocarbon‐based cuticle (C_28_‐C_32_) [24]. Inspired by the biological system, an artificial cuticle is designed to be compositionally similar to its natural counterpart by employing an all‐hydrocarbon OTS–squalane coating on PDMS, as illustrated in Figure 2a. For a robust hydrophobic interface, OTS serves as a key silane coupling agent to introduce chemically grafted hydrophobic 18‐carbon alkyl chains onto the PDMS substrate [27]. To facilitate robust chemical grafting, the PDMS substrate was subjected to air plasma treatment for 2 min, introducing surface hydroxyl groups. Subsequently, the hydroxy‐terminated PDMS was immersed in an OTS solution (1 wt.% in n‐Hexane). It allows the trichlorosilane headgroups of OTS to react with the hydroxyl groups and form stable siloxane linkages through a condensation reaction. However, repetitive droplet impacts can lead to cracking within the highly brittle OTS‐functionalized silica layer (1.3–1.5 GPa) [28, 32]. It is considered to accelerate the formation of diffusion pathways for low molecular weight PDMS, which will be discussed in detail later. Such unwanted PDMS oligomer diffusion inevitably deteriorates the durability of the OTS‐grafted surface, rendering it unsuitable for DEGs subjected to continuous water droplet impact. To address this issue, squalane was infiltrated into the OTS‐functionalized PDMS surface. Squalane (C_30_H_62_) serves as an effective stabilizing agent, highlighted not only for its established biocompatibility in cosmetic applications but also for its exceptional chemical affinity with the OTS layer [33, 34]. To theoretically prove the affinity and stabilization mechanism, the thermodynamic interactions were analyzed using Hansen solubility parameters, as expressed in the following Equation (1) [35, 36]

(1) $${R_{a}}^{2}=4{\left(\delta_{d1}-\delta_{d2}\right)}^{2}+{\left(\delta_{p1}-\delta_{p2}\right)}^{2}+{\left(\delta_{h1}-\delta_{h2}\right)}^{2}$$

where R_a_ is the interaction distance between two substances. A smaller R_a_ value indicates higher solubility and chemical compatibility. δ_d_, δ_p_, and δ_h_ are the dispersion, polar, and hydrogen‐bonding components of the solubility parameter, respectively. The individual components for each material are detailed in Table S2 [33, 36, 37].

The calculated R_a_ value for the squalane‐OTS pair is remarkably low (≈ 0.40). The near‐zero distance indicates that squalane establishes exceptionally strong intermolecular interactions with the octadecyl tails of OTS, driven predominantly by Van der Waals dispersion forces [35]. Simultaneously, the compatibility with PDMS (R_a_ ≈ 4.14) allows squalane to diffuse into the matrix. Consequently, these synergistic interactions enable the infiltrated squalane to stabilize the OTS layer. Specifically, the infused squalane acts as a liquid buffer that mitigates the mechanical stress from repetitive droplet impacts, thereby suppressing the fatigue‐induced micro cracking of the brittle OTS layer. Consequently, this structural protection minimizes the formation of diffusion pathways for low molecular weight oligomers, significantly delaying hydrophobic degradation. Furthermore, the underlying PDMS substrate serves as a stabilizing squalane reservoir. Although surface squalane is gradually removed by water droplet impact, thermodynamic compatibility induces a diffusive flux from the bulk to the OTS surface. Ultimately, the self‐replenishing capability realizes the bio‐inspired design of the artificial cuticle, which overcomes the substrate roughness to ensure efficient uni‐directional droplet guiding along the artificial midrib. In addition to its Aloe‐inspired droplet managing capabilities, the artificial cuticle preserves the high optical transparency, as shown in Figure 2b. The artificial cuticle maintains 97% transmittance at 550 nm wavelength compared to the pristine PDMS. The high transparency is acquired due to the similar refractive index of PDMS and squalane (≈1.44) [38, 39]. Furthermore, the artificial cuticle integrated A‐DEG allows for clear visibility of the red maple leaves background, demonstrating high transmittance in the 650–750 nm wavelength range. Such high optical transparency further enables the hybrid integration of the A‐DEG with photovoltaic (PV) cells without compromising their photoelectric performance, as demonstrated in Figure S3.

To quantitatively confirm the enhanced water repellency, the wetting characteristics of the artificial cuticle were compared with those of pristine PDMS. As presented in Figure 2c, optical images of water droplets were captured on flat surfaces of both pristine PDMS and the Aloe‐inspired artificial cuticle after a pre‐conditioning process involving 3,000 droplet impacts to remove residual bulk oil. The variation of CAH and sliding angle with droplet impact number is summarized in Figure S4. They present static contact angle (SCA), advancing contact angle (ACA), receding contact angle (RCA), and resulting contact angle hysteresis (CAH) with 10 µL water droplets. Moreover, sliding angle measurements with 20 µL water droplets are presented. Detailed contact angle values for all investigated surfaces are provided in Figure S5. As shown in Figure 2d, the droplet on the pristine PDMS exhibited a pinned state (CAH ≈ 87.4°). Conversely, the artificial cuticle displayed enhanced dynamic water repellency, leading to an appropriate state (CAH ≈ 50.6°). The significant difference in hysteresis (∆CAH ≈ 36.8°) indicates that the artificial cuticle lowers surface adhesion and suppresses droplet pinning. In particular, the specific state was designed to ensure continuous electrode contact by suppressing the droplet jumping induced by superhydrophobic surfaces. Instead, it maintains optimized water repellency, achieving stable and uni‐directional guiding along the artificial midrib. Beyond these wetting parameters, the dynamic sliding capability is also essential for minimizing residual water on the A‐DEG surface. To evaluate the capability, sliding angle measurements were conducted as schematically illustrated in Figure S6. While a 20 µL droplet on the pristine PDMS remained pinned even at a 90° tilt, the droplet on the artificial cuticle easily slid off at a lower tilting angle of ≈ 57°. It validates the effective residual water removal potential of the artificial cuticle, which is crucial for the A‐DEG employing a spreading‐sliding droplet mechanism. Furthermore, the sliding angle exhibited an inverse correlation with droplet volume, decreasing from 90° to 40° as the volume increased from 10 to 35 µL, as presented in Figure 2e. Corresponding optical images and detailed sliding angle values for each volume are provided in Figure S7. This behavior occurs because the volume‐dependent gravitational force grows faster than the area‐dependent pinning force. Consequently, these wetting characteristics verify the minimized surface resistance of the artificial cuticle, which is essential for the stable operation of the A‐DEG.

### 2.3 Working Principle and Structural Characteristics of A‐DEG

**FIGURE 3:**
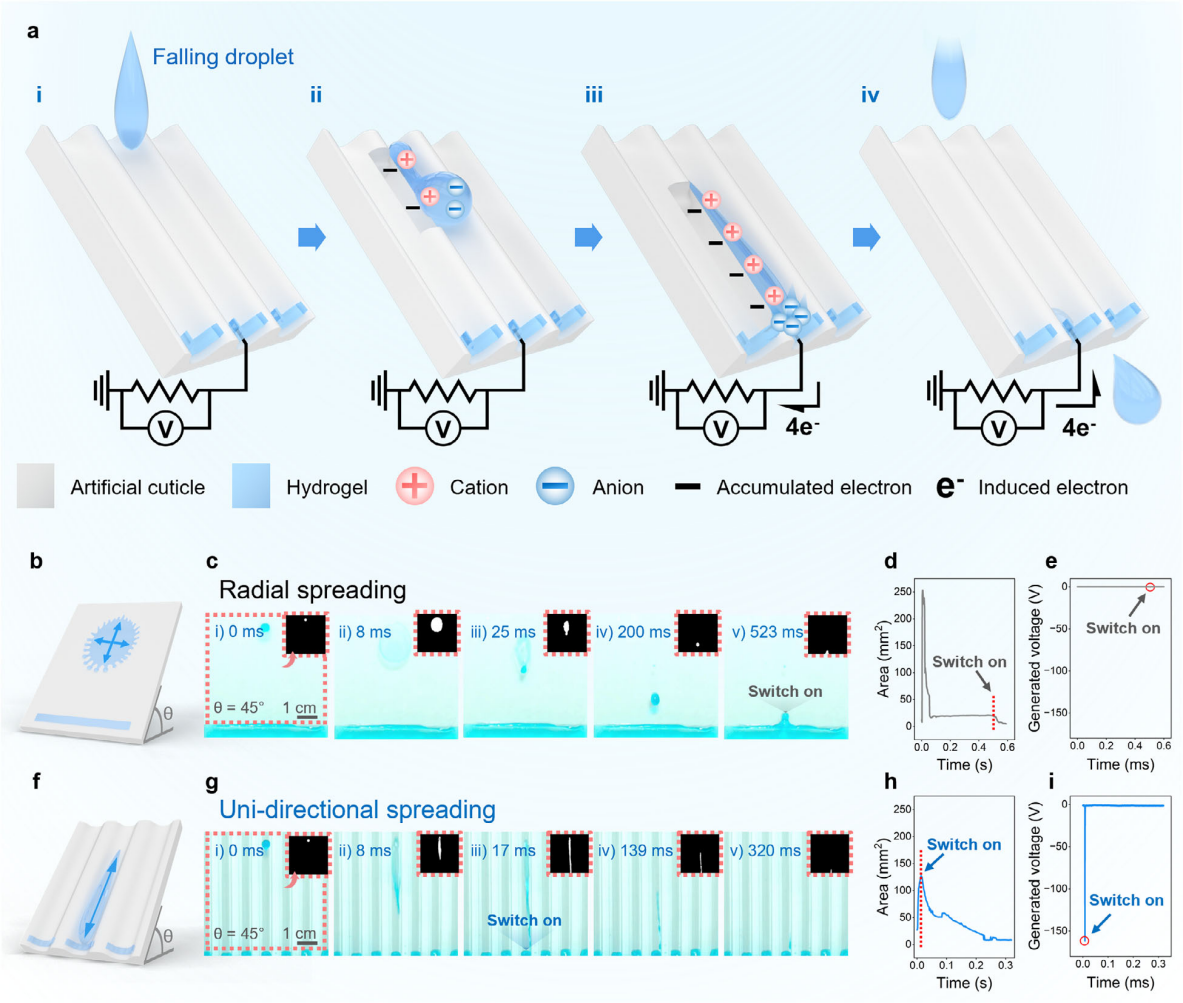
Working principle and structural characteristics of A‐DEG. (a) Schematic illustration of the working principle of A‐DEG with single hydrogel switch electrode. Water droplets falling at a position distant from the electrode (i), initial contact with the surface (ii), uni‐directional spreading that ensures stable contact with the hydrogel switch electrode (iii), and droplet detachment leading to electron transfer (iv). (b) Schematic illustration of radial droplet spreading on conventional DEG. (c) Captured original and binary images of a radially spreading droplet on conventional DEG. (d,e) Insufficient water droplet‐artificial cuticle contact area at maximum spread (d), resulting in unremarkable voltage output (e). (f) Schematic illustration of uni‐directional droplet spreading along A‐DEG channel. (g) Captured original and binary images of uni‐directionally spreading droplet on A‐DEG. (h,i) Effective contact between water droplet and hydrogel electrode at maximum spread (h), generating dramatically enhanced voltage output (i).

Based on the optimized rootward guiding midrib and water‐repellent cuticle, we designed the A‐DEG ensuring efficient uni‐directional droplet spreading and reliable contact with the single hydrogel switch electrode. With this reliable contact, the electrical energy generation is achieved through contact electrification and electrostatic induction, facilitated by the bulk effect. Figure 3a illustrates the detailed working principle of the A‐DEG under a condition where a water droplet falls at a position distant from the hydrogel switch electrode (i). Upon impact (ii), the droplet and the artificial cuticle accumulate equivalent positive charges (cations) and negative charges (electrons) at the interface due to contact electrification. At this stage, these separated charge pairs form an electric double layer (EDL) with a value of capacitance C_cuticle_ shown in Figure S8. Subsequently, the channel structure inspired by the Aloe midrib guides the droplet to spread uni‐directionally toward the hydrogel switch electrode (iii). At contact, an additional EDL forms at the interface between the water droplet and the hydrogel electrode, introducing an interfacial capacitance C_EDL_. It serves as a switch that closes the whole circuit, effectively connecting C_cuticle_ and C_EDL_ in series. As a result, the high electrical potential induced by the charges stored in C_cuticle_ drives an instantaneous flow of electrons through the external circuit. The peak output voltage generated during droplet contact can be expressed as [40]

(2) $$V=\frac{\sigma A_{cuticle}}{C_{EDL}}\frac{R_{Load}}{R_{water}+R_{Load}}$$

where σ is the surface charge density generated at the water–cuticle interface and A_cuticle_ is the effective droplet–sample contact area. This equation indicates that the output voltage scales directly with the interfacial contact area, as a larger contact area enables the accumulation of a greater amount of surface charges prior to discharge. A detailed derivation of Equation (2) is provided in the Supporting Text. Although a high voltage is generated during the process, the energy harvesting is driven by a transient current associated with C_EDL_ formation, thereby preserving the electrochemical stability of the hydrogel without inducing chemical reactions. Finally, owing to the water‐repellent artificial cuticle, the droplet is completely removed without residual water, enabling stable energy harvesting from subsequent droplets (iv).

Compared to the A‐DEG, the structural limitations of the conventional DEG are clearly revealed by high‐speed optical analysis, as shown in Movie S1. As schematically illustrated in Figure 3b, the droplet spreads radially, which results in an inefficient energy collection zone. The high‐speed images in Figure 3c demonstrate that the spreading droplet (≈ 252 mm^2^) rapidly shrinks back to its original shape (t = 25 ms) before reaching the electrode located at a distance of 7 cm. Consequently, Figure 3d,e exhibits that the retracted droplet contacts the electrode (t = 523 ms) with a minimized contact area (≈ 15 mm^2^), generating negligible voltage output (≈ −3 V). In contrast, the A‐DEG enables uni‐directional spreading along the artificial midrib, as schematically illustrated in Figure 3f. High‐speed images in Figure 3g show that the spreading droplet maintains its elongated shape, and contacts the hydrogel switch electrode at t = 17 ms, instantaneously activating the switch at the maximum spreading. As a result, Figure 3h,i demonstrates a clear synchronization between the peak voltage (≈ −169 V) and the maximum contact area (≈ 127 mm^2^). To quantify the performance of A‐DEG, we calculated the energy conversion efficiency (η) for a single droplet impact, representing the conversion from mechanical potential energy to electrical energy, as expressed in the following equation (5) [10]

(3) $$\;\eta =\frac{E_{electrical}}{E_{potential}}=\frac{\int \frac{V{\left(t\right)}^{2}}{R}dt}{mgh}$$

where R is load resistance, m is droplet mass, g is the gravitational acceleration, h is the droplet release height. Following the equation, the electrical energy harvested by the A‐DEG was determined to be 140 nJ, as shown in Figure S9. Also, the mechanical potential energy was calculated as 106 µJ, considering m of 0.054 g, g of 9.81 m/s^2^, and h of 0.2 m. Consequently, the η of the A‐DEG was calculated to be 0.13%

### 2.4 Energy Harvesting Performance on Various Factors of A‐DEG

**FIGURE 4:**
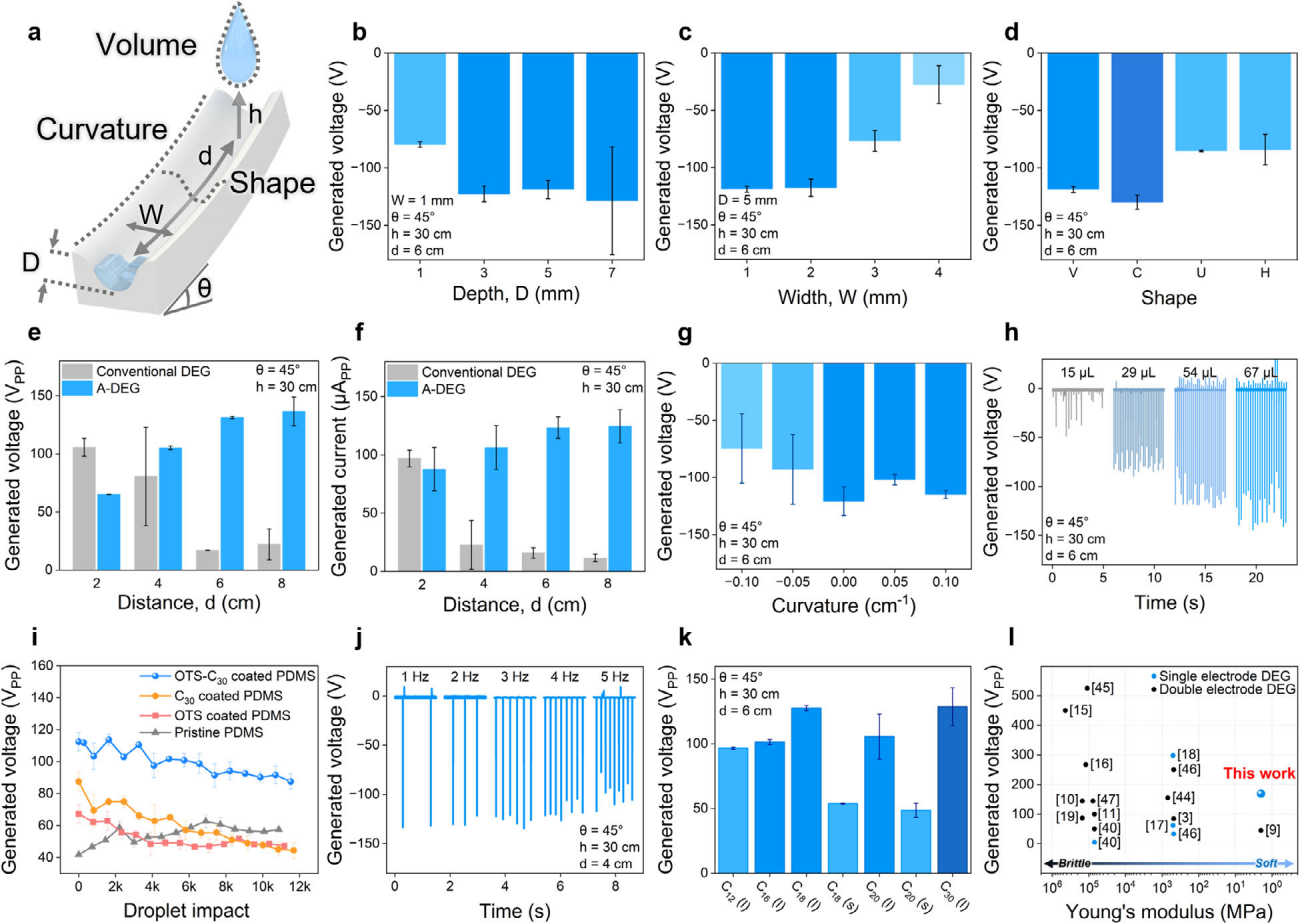
Energy harvesting performance of A‐DEG. (a) Schematic illustration showing key experimental parameters of A‐DEG. (b‐d) Voltage generation on structural design components, including depth (b), width (c), and alphabet‐like shape (d). (e,f) Comparison of voltage (e) and current (f) generation between conventional and Aloe‐inspired DEGs under various distances, highlighting the maximized energy collection zone of A‐DEG. (g) Voltage generation under various curvature, exhibiting flexibility of PDMS based A‐DEG. (h) Voltage generation of A‐DEG as a function of diverse droplet volume (15–67 µL) based on the bulk effect. (i) Comparison of PDMS‐based A‐DEGs with pristine, OTS, C_30_, and OTS–C_30_ coatings in terms of droplet impact durability. (j) Voltage generation of A‐DEG under various droplet impinging frequency. (k) Voltage generation of A‐DEGs with diverse alkane coatings, including liquid (C_12_, C_16_, C_18_, C_20_, C_30_) and solid (C_18_, C_20_) phases. (l) Material–performance comparison of representative droplet electricity generators. Peak voltage versus Young's modulus for single and double‐electrode DEGs.

To maximize the electrical output of the A‐DEG, the influence of key experimental parameters was systematically investigated. Figure 4a illustrates the experimental parameters, including droplet release height (h), electrode distance (d), tilting angle (θ), and bending curvature. In addition, the geometric dimensions of the artificial midrib, which are specifically depth (D), width (W), and cross‐sectional shape, were examined. The detailed definitions for these structural parameters are illustrated in Figure S10. The evaluation criteria were based on the generated voltage, which is directly proportional to the efficiency of uni‐directional droplet spreading and the minimization of residual water on the surface. All droplet experiments were conducted using tap water to emulate practical operating conditions, and the reported voltages are presented as mean ± standard deviation of peak values obtained from five consecutive droplet impacts (n = 5). First, the effect of the channel depth (D) on voltage generation was analyzed. As shown in Figure 4b, increasing D from 1 to 7 mm generally enhanced the voltage output from −79.7 ± 2.4 V to −128.6 ± 46.8 V. It suggests that deeper channels provide higher sidewalls for effective droplet confinement, ensuring stable uni‐directional spreading. However, at D = 7 mm, a significant standard deviation was observed. It occurs because the steep sidewalls interfere with the appropriate droplet landing on the surface, which results in unstable sliding. Therefore, a depth range of 3–5 mm was identified as optimal for generating substantial and stable output. Conversely, the channel width (W) exhibited an inverse relationship with the voltage output, as presented in Figure 4c. The narrower channels (W = 1 and 2 mm) yielded high voltages of −118.7 ± 2.6 V and −117.4 ± 7.5 V, respectively, by guiding droplet spreading uni‐directionally. However, as W increased to 3 and 4 mm, the voltage rapidly dropped to −76.5 ± 9.2 V and −27.6 ± 16.4 V. It is attributed to the wider channel bottoms that cause the droplet to spread radially rather than uni‐directionally. The undesired radial spreading behavior reduces the effective contact area along the longitudinal direction, thereby degrading the electrical performance. Furthermore, the tendency for droplet pinning was found to strongly depend on the channel width and surface patterning. As directly visualized in Movie S2, droplets on wider channels (W = 4 mm) exhibit pronounced pinning at the channel sidewalls, whereas narrow channels (W = 1 mm) enable smooth transport with minimal pinning. Moreover, Figure 4d shows the artificial midrib channel structure by categorizing it into V, C, U, and H shapes. The V‐shape, corresponding to a sharp bottom (W = 1 mm), and the C‐shape (semi‐circular) exhibited superior performance with voltages of −118.7 ± 2.6 V and −129.8 ± 6.3 V, respectively. Both channels effectively guide the droplet to spread uni‐directionally without leaving residual water. In contrast, the U and H shapes, characterized by flatter bottoms, resulted in lower outputs (≈ −85 V) due to inefficient spreading dynamics. Consequently, the C‐type channel was selected as the optimal design and adopted for all subsequent experiments.

With this optimized channel configuration established, the energy harvesting capability of the A‐DEG was compared with that of a conventional DEG. Figure 4e presents the peak‐to‐peak voltage generation at various distances (d) between the droplet impact position and the electrode. At a short distance of d = 2 cm, the conventional DEG yielded a higher output (105.7 ± 7.7 V) than the A‐DEG (65.2 ± 0.1 V). However, a precipitous voltage degradation to negligible levels was observed in the conventional DEG as the distance increased from 4 to 8 cm. In contrast, the A‐DEG demonstrated a progressive enhancement, achieving a voltage of 136.5 ± 12.4 V at d = 8 cm. A similar trend was observed in the peak‐to‐peak current, as shown in Figure 4f. While the output current of the conventional DEG decreased significantly from 96.9 ± 7.2 µA to 11.6 ± 3.3 µA, the A‐DEG exhibited a steady increase from 87.8 ± 18.6 µA to 124.5 ± 14.2 µA as the distance extended to 8 cm. The results highlight that the artificial midrib effectively prevents radial dispersion during transport, thereby maximizing the effective energy collection zone. Figure 4g shows the adaptability of the intrinsic flexible A‐DEG by presenting the voltage generation under various bending curvatures. The detailed bending configurations corresponding to each curvature are visualized in Figure S11. The device maintained sufficient voltage generation across a range of curvatures from −0.10 to 0.10 cm^−1^. Notably, under concave bending conditions (curvature ≥ 0 cm^−1^, resembling a cycloid curve), the device exhibited robust performance with voltage magnitudes exceeding 75 V. However, under convex bending conditions (curvature < 0 cm^−1^, resembling a hill ridge), a reduction in voltage was observed (−74.6 ± 30.3 V at −0.10 cm^−1^). This instability arises from the increased probability of the sliding droplet lifting off from the convex surface, thereby failing to contact the switch electrode. Nevertheless, the overall performance highlights the potential of the A‐DEG for flexible energy harvesting applications. Moreover, the effect of droplet volume on the electrical output was investigated, as shown in Figure 4h. The generated voltage was measured for droplet volumes of 15, 29, 54, and 67 µL. At a small volume of 15 µL, the droplet barely contacts the hydrogel electrode due to insufficient uni‐directional spreading, resulting in unstable and low‐voltage output. As the volume increased to 29 µL and above, stable contact was established. Moreover, the voltage output exhibited a proportional increase with droplet volume. The enhancement is attributed to the enlarged contact area at the droplet‐surface interface, which facilitates a stronger bulk effect.

To evaluate the performance of the artificial cuticle, the electrical output of the A‐DEG was measured across four different surface modifications, as presented in Figure S12. The modifications include pristine, OTS‐coated, squalane (C_30_) coated, and OTS–C_30_ coated PDMS. The A‐DEG utilizing pristine PDMS yielded the lowest output (≈ −45 V) due to its insufficient water repellency, which hinders charge transfer. The working principle on such surface is detailed in Figure S13. While the OTS coating improved hydrophobicity, it resulted in a moderate voltage output of ≈ −75 V. The C_30_ coated A‐DEG enhanced the voltage to ≈ −100 V. Remarkably, the OTS–C_30_ coating, which is designated as the artificial cuticle, achieved the highest voltage of ≈ −125 V. The superior performance is attributed to the synergistic effect of the water‐repellent artificial cuticle and the artificial channeling midrib. To ensure the sustained operability of the A‐DEG under practical conditions such as continuous rainfall, the persistence of electrical output was intentionally evaluated against repetitive droplet impacts onto a single point. The durability of these surface modifications was further examined by monitoring voltage variations at over 10,000 successive droplet impacts, as shown in Figure 4i. The A‐DEG with pristine PDMS maintained a relatively low voltage output (41.7 ± 0.6 V to 57.0 ± 1.4 V) throughout the cycles, confirming its unsuitable wetting characteristics for the artificial midrib. The OTS‐coated A‐DEG exhibited an initial voltage of 67.3 ± 5.7 V, but the output degraded to 69% of its initial value. The degradation indicates the vulnerability of the OTS‐functionalized PDMS surface to consecutive water droplet impacts. Similarly, the C_30_ coated A‐DEG showed a notable voltage drop to 50% of its initial value (87.5 ± 6.1 V) as the surface oil was rapidly washed away. Conversely, the A‐DEG employing the artificial cuticle exhibited further enhanced durability, maintaining a stable output for the first 3,000 cycles. Although a gradual decrease was observed thereafter, it retained 78% of its initial high voltage (112.5 ± 5.7 V) even after 10 000 impacts, outperforming the charge‐saturated pristine PDMS‐based A‐DEG. Notably, as further demonstrated in Figure S14, increasing the diffusion time of squalane into the OTS‐functionalized PDMS effectively enhances both the voltage magnitude and its long‐term durability under successive droplet impacts. In particular, the A‐DEG with a 6‐h diffusion exhibits an increased voltage output reaching 126% of its initial value even after 11,000 droplet impacts. Figure 4j presents the dependence of voltage generation on droplet impinging frequency. The A‐DEG maintained a consistent voltage output (≈ −130 V) from 1 to 3 Hz. However, at frequencies exceeding 4 Hz, a gradual decrease in voltage was observed. The voltage reduction is attributed to the insufficient interval between droplet impacts. It causes the subsequent droplet to overlap with the preceding one before its complete removal, thereby disrupting the efficient charge transfer cycles. Finally, the effect of alkane chain length and phase on the electrical output was analyzed to optimize the artificial cuticle composition, as summarized in Figure 4k. Seven types of alkanes were used, including liquid phases (C_12_, C_16_, C_18_, C_20_, C_30_) and solid phases (C_18_, C_20_). Their melting points are detailed in Table S3 [41]. Generally, liquid‐phase alkanes yielded higher voltages than solid counterparts, as solid‐phase alkanes induce surface crystallization that leads to pinning and voltage reduction. Among the liquid alkanes, the voltage increased with chain length starting from C_18_. However, C_20_ exhibited a real‐time voltage drop due to phase transition into a solid state upon droplet impact. Consequently, squalane (C_30_) was identified as the most suitable coating material for the A‐DEG. Squalane not only yields enhanced electrical output but also presents superior biocompatibility compared with shorter‐chain alkanes (< C_20_) [34, 42, 43]. The biocompatibility ensures that potential oil leakage from the A‐DEG remains ecologically harmless. Furthermore, to evaluate the power generation capability of the optimized A‐DEG, the output voltage and instantaneous peak power were measured under varying external load resistances, as shown in Figure S15. The A‐DEG exhibited a maximum peak power of 2.8 mW at a matched impedance of 500 kΩ, demonstrating its suitability for driving low‐power electronics. In addition, to compare the electrical performance of A‐DEG against previously reported DEGs, the peak voltage output is compared with representative literature values as a function of the Young's modulus of the device material, as shown in Figure 4l [3, 9, 10, 11, 15, 16, 17, 18, 19, 40, 44, 45, 46, 47]. This comparison shows that the A‐DEG occupies a distinct performance regime by combining high voltage output with exceptional mechanical softness and a single‐electrode architecture. Notably, unlike conventional DEGs in which energy harvesting is largely confined to the local droplet impact region, the A‐DEG features an expanded energy collection zone enabled by the artificial midrib‐guided surface. Therefore, when considering voltage generation over a range of droplet–electrode distances rather than a single impact event, the A‐DEG is expected to exhibit a substantially enhanced overall energy harvesting capability compared to previously reported DEGs.

### 2.5 Demonstration of Maximized Energy Collection Zone in A‐DEG

**FIGURE 5:**
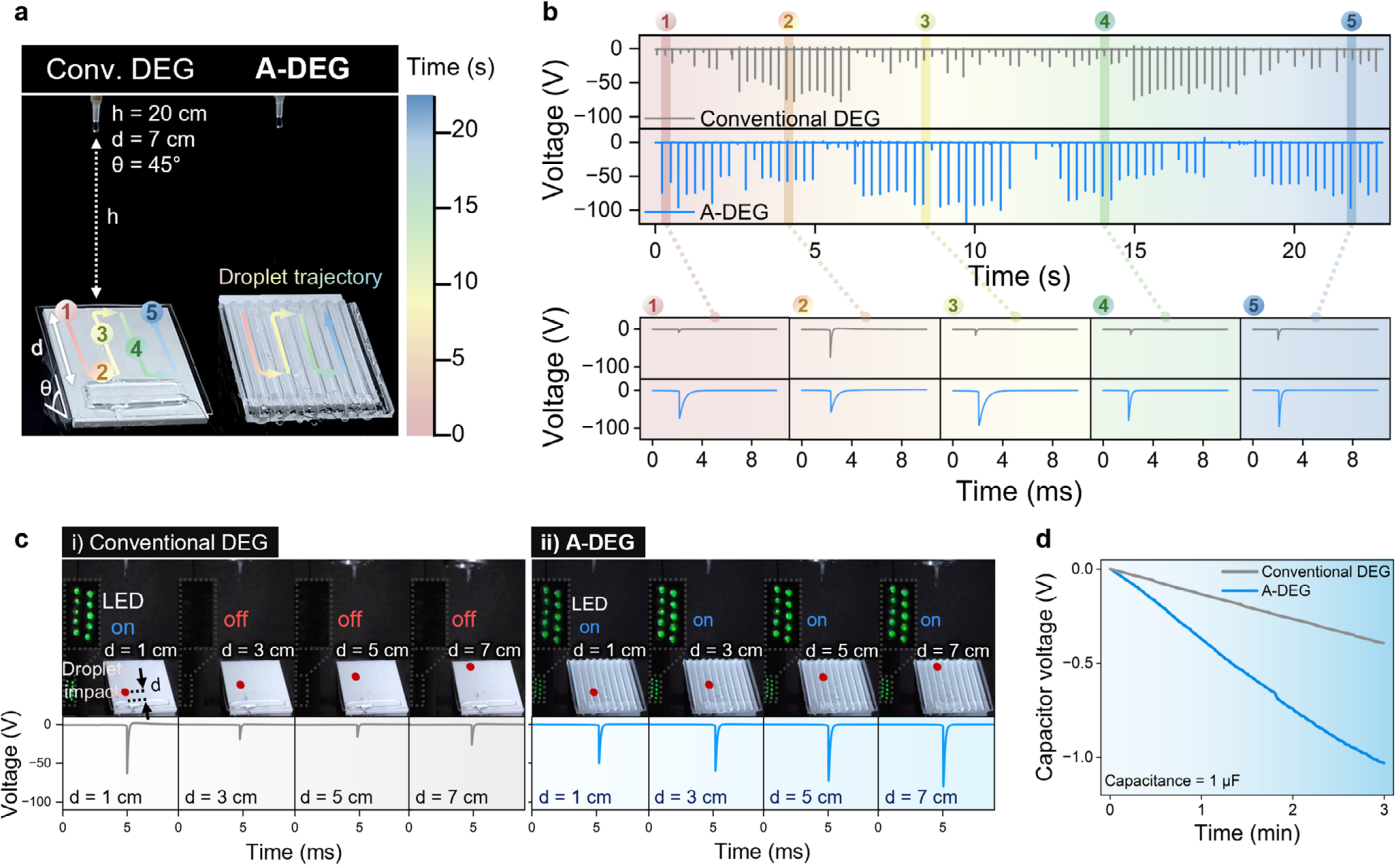
Validation of maximized energy collection zone in A‐DEG. (a) Falling droplet along predefined droplet trajectory onto both DEGs. (b) Voltage generation of conventional DEG (grey) and A‐DEG (blue) along droplet trajectory. A‐DEG exhibits remarkably increased energy collection at diverse positions compared to the conventional DEG. (c) Energy harvesting capability visualized through LED illumination of conventional and Aloe‐inspired DEGs at various distances (1–7 cm) from droplet impact position to hydrogel electrode. Continuous LED illumination in A‐DEG is observed across all distances, unlike the limited LED illumination in conventional DEG. (d) Capacitor charging behavior of conventional and Aloe‐inspired DEG under artificial rainfall conditions, where A‐DEG delivers over twice the charge of conventional DEG.

To prove the effectiveness of the maximized energy collection zone provided by the artificial midrib and cuticle, dynamic rainfall conditions were emulated using a programmable robotic arm. As illustrated in Figure 5a, the robotic arm moved a droplet nozzle along a predefined trajectory (points 1 through 5), dispensing droplets at a frequency of 4 Hz onto both the conventional DEG and the A‐DEG. Figure 5b presents the real‐time voltage generation corresponding to the droplet impact positions along this trajectory. The conventional DEG (grey) generated functional voltage peaks (≈ −75 V) only when droplets landed in close proximity to the electrode (point 2). At all other positions (1, 3, 4, and 5), the conventional DEG produced suppressed output (0 to −40 V), as the radially spreading droplets failed to maintain a sufficient contact area upon bridging the electrode. In contrast, the A‐DEG (blue) exhibited consistent and high voltage peaks (≈ −100 V) across various impact points. It demonstrates that the artificial midrib facilitates effective droplet transport to the electrode from most positions on the surface. The compared behaviors clearly highlight that the A‐DEG successfully overcomes the spatial limitations of conventionally designed DEG, realizing a truly maximized energy harvesting capability. To provide an intuitive visualization of this capability, an LED illumination test was conducted at distances (d) ranging from 1 to 7 cm from the electrode, as shown in Figure 5c and Movie S3. The specific circuit configuration used for this demonstration is illustrated in Figure S16. The conventional DEG activated the LEDs only at the shortest distance (d = 1 cm) and failed to harvest sufficient energy to light up the LEDs at distances of 3 cm or greater. Conversely, the A‐DEG successfully illuminated the commercial LEDs at all examined distances (d = 1, 3, 5, 7 cm), demonstrating its reliable operation regardless of the droplet impact position. The result visually validates the superior energy harvesting efficiency of the A‐DEG enabled by the uni‐directional spreading mechanism. The practical efficiency was quantified by charging a capacitor under dynamic conditions. Figure 5d shows the charging behavior of a 1 µF capacitor under an artificial rainfall environment with randomly distributed droplet impacts, as shown in Movie S4. Under identical conditions, the conventional DEG charged the capacitor to approximately −0.39 V within 3 min, whereas the A‐DEG charged the same capacitor to an enhanced voltage of approximately −1.03 V over the same duration. The substantial difference indicates that the A‐DEG effectively converts the kinetic energy of falling droplets into electrical energy, whereas the conventional DEG fails to harvest energy from droplets landing outside its limited active zone. Consequently, the A‐DEG demonstrates an efficient energy harvesting potential in realistic environments with its maximized energy collection zone.

## 3 Conclusions

In summary, A‐DEG successfully addressed the inherent spatial limitations of conventional droplet electricity generators (DEGs) through the replication of the structural and chemical survival strategies of Aloe vera. By synergistically integrating a curvilinear artificial midrib with a hydrocarbon‐based artificial cuticle, the A‐DEG realizes a mechanism for uni‐directionally spreading droplet transport. The approach effectively maximizes its energy collection zone, decoupling device performance from the spatial stochasticity of natural precipitation. Furthermore, the OTS–squalane composite layer, which is designed based on thermodynamic affinity, provides enhanced robustness against repetitive hydraulic stress, overcoming the durability issues commonly associated with brittle silane‐based modifications. Additional studies will extend this approach by examining coupled thermodynamic, interfacial, and droplet‐induced dynamic behaviors under realistic operating conditions. Consequently, the A‐DEG demonstrates a remarkable 236% enhancement in charge output compared to conventional DEG, and maintains stable performance even under dynamic rainfall conditions. The position‐independent A‐DEG decouples electrical output from droplet impact location by enabling guided droplet transport toward the switch electrode across a maximized energy collection zone. Building on this platform, future studies that quantitatively link channel geometry and in situ droplet–surface contact evolution with electrical output will further strengthen the design guidelines needed to translate scalable droplet energy harvesting to real‐world operating conditions.

## 4 Experimental Section

### 4.1 Materials

To fabricate the complex‐structured A‐DEG, polydimethylsiloxane (PDMS; Dow Inc., Sylgard 184) was adopted as the elastomer substrate. For the hydrogel electrode, acrylamide (AAm; Sigma, A8887), and N,N′‐methylenebis(acrylamide) (MBAAm; Sigma, M7279) were used as the monomer and crosslinker, respectively. Ammonium persulfate (APS; Sigma, A3678), and N,N,N′,N′‐tetramethylethylenediamine (TEMED; Sigma, T9281) were added as the thermal initiator and accelerator. Lithium chloride (LiCl; Sigma, 310468) served as the ionic charge carrier. Benzophenone (Sigma, B9300) was used as a surface photo‐initiator for UV‐induced chemical bonding. For hydrophobic surface modification, octadecyltrichlorosilane (OTS; Sigma, 104817) and n‐hexane (Samchun Chemicals, H0113) were used. The water‐repellent capability was further enhanced by covering the A‐DEG surface with various alkane materials, including n‐dodecane (Kanto Chemicals, 10500‐00), n‐hexadecane (Sigma, H6703), n‐octadecane (Sigma, 0652), n‐eicosane (Thermo Scientific, A13853.22), and squalane (Sigma, 234311).

### 4.2 Preparation of Hydrogel Precursor Solution

The ionic hydrogel solution was prepared by dissolving AAm as monomer and lithium chloride as charge carrier in deionized water to form 4 and 16 M aqueous solutions, respectively. The cross‐linker MBAAm (0.05 wt.%), the initiator APS (0.7 wt.%), and the accelerator TEMED (0.35 wt.%) were sequentially added. All components were calculated relative to the weight of the AAm monomer, then subsequently mixed and degassed. Notably, although hydrogels are typically susceptible to dehydration, the continuous water supply from droplet impacts during A‐DEG operation naturally replenishes the hydrogel, effectively eliminating evaporation concerns.

### 4.3 Fabrication of A‐DEG Prior to Surface Modification

First, a custom‐designed mold for PDMS was fabricated using a high‐resolution stereolithography (SLA) printer (Formlabs, Form4) with clear resin. To eliminate unreacted residual photo‐initiators, the printed mold was post‐cured in a UV crosslinker (Formlabs, Form cure) at 70°C for 4 h. To fabricate structured PDMS, a PDMS solution was prepared by mixing base and curing agent (10:1 by weight) and poured into the mold (Figure S17). The solution was cured at 80°C for 4 h in an oven (Changshin Science, C‐DOD3), and the cured PDMS with the wanted structure was gently peeled off from the mold. Subsequently, the cured PDMS was immersed in benzophenone (BP) solution (15 wt.% in ethanol) for 10 min and rinsed with pure ethanol to remove residual BP. Then, the hydrogel solution was poured on the bottom surface of BP‐treated PDMS, and irradiated with 365 nm UV light for 5 min. Through covalent bonding between hydrogel and PDMS, pre‐modified A‐DEG with a hydrogel electrode was fabricated.

### 4.4 Surface Modification of A‐DEG for Water‐repellent Layer

The A‐DEG surface was treated with air plasma (Harrick Plasma, PDC‐002‐HP) for 2 min to convert methyl groups (Si‐CH_3_) into silanol groups (Si‐OH). The plasma treated A‐DEG was immersed in octadecyltrichlorosilane (OTS) solution (1 wt.% in n‐hexane) for 1 min. Covalent Si‐O‐Si bonds were formed at the PDMS‐OTS interface through a condensation reaction between silanol groups on PDMS and trichlorosilane groups of OTS. After rinsing with n‐hexane to remove residual OTS, the sample was heat‐treated at 80°C for 30 min. Finally, lubricating fluid such as squalane (C_30_H_62_) was applied onto the OTS‐functionalized surface at 65°C for 2 h to construct a stable water‐repellent layer.

### 4.5 Data Measurement

The output voltage and current of A‐DEG were measured using an oscilloscope (Tektronix, MDO34, 1 MΩ) equipped with a voltage probe (Tektronix, TPP0850, 40 MΩ) and a low‐noise current preamplifier (Stanford research system, SR570), respectively. To precisely control the droplet impact location, a syringe pump (New Era Pump Systems Inc., NE‐300) and a robotic arm (Universal Robots, UR3e) were used. All experiments were conducted at room temperature and a relative humidity of 40%–45%. The reported electrical outputs are presented as mean ± standard deviation based on peak values extracted from five consecutive droplet events under identical conditions (n = 5) with tap water droplets. For the micrograph of Aloe leaf structure shown in Figure 1b, an Aloe leaf was sliced at an angle of 40–45° and imaged using an optical microscope (Keyence, VHX‐950F). The optical transmittance of the artificial cuticle and pristine PDMS, prepared as flat samples, was measured using a UV–vis spectrometer (V‐650, Jasco, Easton, MD, USA) in the wavelength range of 300–800 nm. In addition, a commercial Arduino‐compatible photovoltaic (PV) cell was integrated with the A‐DEG to demonstrate the optical transparency of the device under an illumination intensity of 1000 lx. To analyze the effect of surface modification on wettability, contact and sliding angles were measured on flat surfaces with and without surface treatment by a contact angle analyzer (Femtobiomed, Smart Drop). The static, advancing, and receding contact angles were obtained via the sessile drop method with 10 µL water droplets (Figure 2b). A high‐speed camera (Kron Tech., CR21‐1.0‐32C) was used to compare the artificial rootward‐structured surface of A‐DEG with the flat surface of conventional DEG, as shown in Figure 3c,g. High‐speed videos in Movie S1 were recorded at 2,142 fps, whereas Movie S2 was recorded at 30 fps.

## Author Contributions

G.L. performed conceptualization, investigation, methodology, formal analysis, and writing. E.K. performed methodology, validation, and visualization. K.C. performed the investigation and writing. M.S. performed validation. S.J. performed the methodology. D.C. performed validation. D.S. performed formal analysis. M.L. performed methodology, supervision, wrote, reviewed, and edited the final manuscript. Y.L. performed conceptualization, supervision, wrote, reviewed, and edited the final manuscript.

## Conflicts of Interest

The authors declare no conflicts of interest.

## Supporting information




**Supporting File 1**: adma72558‐sup‐0001‐SuppMat.docx.

**Supporting File 2**: adma72558‐sup‐0002‐MovieS1.mp4.

**Supporting File 3**: adma72558‐sup‐0003‐MovieS2.mp4.

**Supporting File 4**: adma72558‐sup‐0004‐MovieS3.mp4.

**Supporting File 5**: adma72558‐sup‐0005‐MovieS4.mp4.

## Data Availability

The data that support the findings of this study are available in the supplementary material of this article.

## References

[1] C. Feng , M. Hu , J. Alharbi , M. Rueping , and H. Zhang , “Bridging Scales in Solar‐Driven Water Splitting: Pathways to System Integration,” Advanced Materials 38 (2025): 06690. 10.1002/adma.202506690 41030188

[2] S. Cho , D. Lee , S. Jang , et al., “Physical Intelligence‐based Working Mode Adaptable Triboelectric Nanogenerator for Effective Wind Energy Harvesting in Broad Range,” Nano Energy 113 (2023): 108608, 10.1016/j.nanoen.2023.108608.

[3] S. Jang , S. A. Shah , J. Lee , et al., “Beyond Metallic Electrode: Spontaneous Formation of Fluidic Electrodes from Operational Liquid in Highly Functional Droplet‐Based Electricity Generator,” Advanced Materials 36 (2024): 2403090, 10.1002/adma.202403090. 38695508

[4] S. Hwang , D. Jang , B. Lee , et al., “All Direct Ink Writing of 3D Compliant Carbon Thermoelectric Generators for High‐Energy Conversion Efficiency,” Advanced Energy Materials 13 (2023): 2204171, 10.1002/aenm.202204171.

[5] Y. Lee , W. J. Song , Y. Jung , et al., “Ionic Spiderwebs,” Science Robotic 5 (2020): aaz5405. 10.1126/scirobotics.aaz5405 33022609

[6] Y. Lee , S. Lim , W. J. Song , et al., “Triboresistive Touch Sensing: Grid‐Free Touch‐Point Recognition Based on Monolayered Ionic Power Generators,” Advanced Materials 34 (2022): 2108586, 10.1002/adma.202108586. 35245965

[7] D. Choi , Y. Lee , Z.‐H. Lin , et al., “Recent Advances in Triboelectric Nanogenerators: from Technological Progress to Commercial Applications,” ACS Nano 17 (2023): 11087–11219, 10.1021/acsnano.2c12458. 37219021 PMC10312207

[8] W. J. Song , Y. Lee , Y. Jung , et al., “Soft Artificial Electroreceptors for Noncontact Spatial Perception,” Science advances 7 (2021): abg9203. 10.1126/sciadv.abg9203 PMC8612677 34818043

[9] S. Jang , S. Lee , S. A. Shah , et al., “Hydrogel‐Based Droplet Electricity Generators: Intrinsically Stretchable and Transparent for Seamless Integration in Diverse Environments,” Advanced Functional Materials 35 (2025): 2411350, 10.1002/adfm.202411350.

[10] W. Xu , H. Zheng , Y. Liu , et al., “A Droplet‐Based Electricity Generator with High Instantaneous Power Density,” Nature 578 (2020): 392–396, 10.1038/s41586-020-1985-6. 32025037

[11] Q. Zhang , Y. Li , H. Cai , et al., “A Single‐Droplet Electricity Generator Achieves an Ultrahigh Output over 100 V without Pre‐Charging,” Advanced Materials 33 (2021): 2105761, 10.1002/adma.202105761. 34655116

[12] X. Chen , D. Goodnight , Z. Gao , et al., “Scaling up Nanoscale Water‐Driven Energy Conversion into Evaporation‐Driven Engines and Generators,” Nature Communications 6 (2015): 7346, 10.1038/ncomms8346. PMC4490384 26079632

[13] Z. Zhang , X. Li , J. Yin , et al., “Emerging Hydrovoltaic Technology,” Nature Nanotechnology 13 (2018): 1109–1119, 10.1038/s41565-018-0228-6. 30523296

[14] J. Zhang , S. Lin , M. Zheng , and Z. L. Wang , “Triboelectric Nanogenerator as a Probe for Measuring the Charge Transfer between Liquid and Solid Surfaces,” ACS Nano 15 (2021): 14830–14837, 10.1021/acsnano.1c04903. 34415141

[15] D. C. Nguyen , M. C. Nguyen , D. T. Pham , et al., “Field Effect Enhanced Electric Double Layer for High‐output Droplet Energy Harvester,” Nano Energy 134 (2025): 110560, 10.1016/j.nanoen.2024.110560.

[16] X. Xu , P. Li , Y. Ding , et al., “Droplet Energy Harvesting Panel,” Energy & Environmental Science 15 (2022): 2916–2926, 10.1039/D2EE00357K.

[17] N. Zhang , H. Gu , K. Lu , et al., “A Universal Single Electrode Droplet‐based Electricity Generator (SE‐DEG) for Water Kinetic Energy Harvesting,” Nano Energy 82 (2021): 105735, 10.1016/j.nanoen.2020.105735.

[18] J. Meng , L. Zhang , H. Liu , et al., “A New Single‐Electrode Generator for Water Droplet Energy Harvesting with A 3 mA Current Output,” Advanced Energy Materials 14 (2024): 2303298, 10.1002/aenm.202303298.

[19] Y. Li , Y. Zhang , J. Zhang , et al., “A Transparent Droplet‐Based Electricity Generator Utilizing Patterned Wetting Surfaces,” ACS Applied Materials & Interfaces 17 (2025): 42205–42214, 10.1021/acsami.5c08324. 40650632

[20] X. Xu , Q. Wang , and E. Hirata , “Precipitation Partitioning and Related Nutrient Fluxes in a Subtropical Forest in Okinawa, Japan,” Annals of Forest Science 62 (2005): 245–252, 10.1051/forest:2005016.

[21] C. Yuan , G. Gao , and B. Fu , “Comparisons of Stemflow and Its Bio‐/Abiotic Influential Factors between Two Xerophytic Shrub Species,” Hydrology and Earth System Sciences 21 (2017): 1421–1438, 10.5194/hess-21-1421-2017.

[22] L. Suriati , I. M. S. Utama , B. A. Harsojuwono , and I. B. W. Gunam , “Effect of Additives on Surface Tension, Viscosity, Transparency and Morphology Structure of Aloe Vera Gel‐Based Coating,” Frontiers in Sustainable Food Systems 6 (2022): 831671, 10.3389/fsufs.2022.831671.

[23] T. H. Yeats and J. K. Rose , “The Formation and Function of Plant Cuticles,” Plant Physiology 163 (2013): 5–20, 10.1104/pp.113.222737. 23893170 PMC3762664

[24] R. C. Racovita , C. Peng , T. Awakawa , I. Abe , and R. Jetter , “Very‐Long‐Chain 3‐Hydroxy Fatty Acids, 3‐Hydroxy Fatty Acid Methyl Esters and 2‐Alkanols from Cuticular Waxes of Aloe Arborescens Leaves,” Phytochemistry 113 (2015): 183–194, 10.1016/j.phytochem.2014.08.005. 25200334

[25] H. Silva , S. Sagardia , M. Ortiz , et al., “Relationships between Leaf Anatomy, Morphology, and Water Use Efficiency in Aloe vera (L) Burm f. as a Function of Water Availability,” Revista Chilena de Historia Natural 87 (2014): 13, 10.1186/s40693-014-0013-3.

[26] Y. Lee , S. H. Cha , Y.‐W. Kim , D. Choi , and J.‐Y. Sun , “Transparent and Attachable Ionic Communicators Based on Self‐cleanable Triboelectric Nanogenerators,” Nature Communications 9 (2018): 1804, 10.1038/s41467-018-03954-x. PMC5935721 29728600

[27] G. S. Ferguson , M. K. Chaudhury , H. A. Biebuyck , and G. M. Whitesides , “Monolayers on Disordered Substrates: Self‐Assembly of Alkyltrichlorosilanes on Surface‐Modified Polyethylene and Poly(dimethylsiloxane),” Macromolecules 26 (1993): 5870–5875, 10.1021/ma00074a007.

[28] H. Hillborg and U. Gedde , “Hydrophobicity Recovery of Polydimethylsiloxane after Exposure to Corona Discharges,” Polymer 39 (1998): 1991–1998.

[29] J. Kim , M. K. Chaudhury , M. J. Owen , and T. Orbeck , “The Mechanisms of Hydrophobic Recovery of Polydimethylsiloxane Elastomers Exposed to Partial Electrical Discharges,” Journal of Colloid and Interface Science 244 (2001): 200–207, 10.1006/jcis.2001.7909. 16055136

[30] T.‐S. Wong , S. H. Kang , S. K. Tang , et al., “Bioinspired Self‐repairing Slippery Surfaces with Pressure‐Stable Omniphobicity,” Nature 477 (2011): 443–447, 10.1038/nature10447. 21938066

[31] D. Yoo , S. Jang , S. Cho , D. Choi , and D. S. Kim , “A Liquid Triboelectric Series,” Advanced Materials 35 (2023): 2300699, 10.1002/adma.202300699. 36947827

[32] S. Béfahy , P. Lipnik , T. Pardoen , et al., “Thickness and Elastic Modulus of Plasma Treated PDMS Silica‐Like Surface Layer,” Langmuir 26 (2010): 3372–3375, 10.1021/la903154y. 19947617

[33] D. W. Van Krevelen and K. T. Nijenhuis , Properties of Polymers: Their Correlation with Chemical Structure; Their Numerical Estimation and Prediction From Additive Group Contributions (Elsevier, 2009).

[34] S.‐K. Kim and F. Karadeniz , “Biological Importance and Applications of Squalene and Squalane,” Advances in Food and Nutrition Research 65 (2012): 223–233. 22361190 10.1016/B978-0-12-416003-3.00014-7

[35] Y. Agata and H. Yamamoto , “Determination of Hansen Solubility Parameters of Ionic Liquids Using Double‐sphere Type of Hansen Solubility Sphere Method,” Chemical Physics 513 (2018): 165–173, 10.1016/j.chemphys.2018.04.021.

[36] C. M. Hansen , Hansen Solubility Parameters: A User's Handbook (CRC press, 2007), 10.1201/9781420006834.

[37] T. Uragami , I. Sumida , T. Miyata , T. Shiraiwa , H. Tamura , and T. Yajima , “Pervaporation Characteristics in Removal of Benzene from Water through Polystyrene‐Poly (dimethylsiloxane) IPN Membranes,” Materials Sciences and Applications 2 (2011): 169.

[38] K. Lal , N. Tripathi , and G. P. Dubey , “Densities, Viscosities, and Refractive Indices of Binary Liquid Mixtures of Hexane, Decane, Hexadecane, and Squalane with Benzene at 298.15 K,” Journal of Chemical & Engineering Data 45 (2000): 961–964, 10.1021/je000103x.

[39] F. Schneider , J. Draheim , R. Kamberger , and U. Wallrabe , “Process and Material Properties of Polydimethylsiloxane (PDMS) for Optical MEMS,” Sensors and Actuators A: Physical 151 (2009): 95–99, 10.1016/j.sna.2009.01.026.

[40] K. Chaithaweep , U. Pharino , S. Pongampai , et al., “High‐Performance Droplet‐Based Triboelectric Nanogenerators: a Comparison of Device Configuration and Operating Parameters,” Advanced Materials Technologies 10 (2025): 2401870, 10.1002/admt.202401870.

[41] W. M. Haynes , CRC Handbook of Chemistry and Physics (CRC press, 2016), 10.1201/9781315380476.

[42] R. Babu , A. Chatterjee , and M. Singh , “Assessment of Skin Irritation and Molecular Responses in Rat Skin Exposed to Nonane, Dodecane and Tetradecane,” Toxicology Letters 153 (2004): 255–266, 10.1016/j.toxlet.2004.04.036. 15451557

[43] R. Mallampati , R. R. Patlolla , S. Agarwal , et al., “Evaluation of EpiDerm Full Thickness‐300 (EFT‐300) as an in Vitro Model for Skin Irritation: Studies on Aliphatic Hydrocarbons,” Toxicology in Vitro 24 (2010): 669–676, 10.1016/j.tiv.2009.08.019. 19720135 PMC2947439

[44] L. Wang , W. Li , Y. Song , et al., “Monolithic Integrated Flexible yet Robust Droplet‐Based Electricity Generator,” Advanced Functional Materials 32 (2022): 2206705, 10.1002/adfm.202206705.

[45] C. Wang , J. Wang , P. Wang , et al., “High‐Entropy Ceramics Enhanced Droplet Electricity Generator for Energy Harvesting and Bacterial Detection,” Advanced Materials 36 (2024): 2400505, 10.1002/adma.202400505. 38782490

[46] X. Li , X. Ning , L. Li , et al., “Performance and Power Management of Droplets‐based Electricity Generators,” Nano Energy 92 (2022): 106705, 10.1016/j.nanoen.2021.106705.

[47] X. Wang , S. Fang , J. Tan , et al., “Dynamics for Droplet‐based Electricity Generators,” Nano Energy 80 (2021): 105558, 10.1016/j.nanoen.2020.105558.

