# Supplementary material for: Droplet Electricity Generators With Maximized Energy Collection Zone Enabled by Aloe‐Inspired Midrib and Cuticle
Source: Adv Mater. 2026 Mar 12;38(39):e23637. doi: 10.1002/adma.202523637 (PMC13361258; doi:10.1002/adma.202523637)
Supplement: Supplementary file 1 — Supporting File 1: adma72558‐sup‐0001‐SuppMat.docx. [file ADMA-38-e23637-s005.docx]

Supporting Information

**Droplet electricity generators with maximized energy collection zone enabled by aloe-inspired midrib and cuticle**

Gibeom Lee^a^, Eunbyeol Kim^b^, Kyongtae Choi^a^, Minjun Song^a^, Sunmin Jang^a^, Dongwhi Choi^a^, Donghyun Seo^c^, Min-Gyu Lee^d^*, Younghoon Lee^a^*

**Supplementary Text**

Derivation of the Peak Output Voltage during Droplet Contact

During the droplet–electrode contact, the electrical response can be described by an RC discharge model, where the accumulated interfacial charge Q(t) is realized through the equivalent resistance R_eq_ and capacitance C_eq_. Applying Kirchhoff’s voltage law to the loop yields

$$\begin{aligned} V_{R}\left( t \right)+V_{C}\left( t \right)=R_{eq}I\left( t \right)+\frac{Q\left( t \right)}{C_{eq}}=0\#(S1) \end{aligned}$$

Rearranging Eq. (S1) gives

$$\begin{aligned} I\left( t \right)= -\frac{Q\left( t \right)}{R_{eq}C_{eq}}\to\frac{I\left( t \right)}{Q\left( t \right)}=-\frac{1}{R_{eq}C_{eq}}\#(S2) \end{aligned}$$

Using the constitutive relation I(t) = dQ(t)/dt, Eq. (S2) becomes

$$\begin{aligned} \frac{1}{Q\left( t \right)}dQ= -\frac{1}{R_{eq}C_{eq}}dt\#(S3) \end{aligned}$$

Integrating both sides from t = 0 (with Q(0) = Q_0_) to an arbitrary time t (with Q(t)),

$$\begin{aligned} \int_{Q_{0}}^{Q_{t}} \frac{1}{Q}dQ=-\int_{0}^{t} \frac{1}{R_{eq}C_{eq}}dt, \\ \ln\left( \frac{Q\left( t \right)}{Q_{0}} \right)=- \frac{t}{R_{eq}C_{eq}}\#(S4) \end{aligned}$$

Therefore, the charge evolution follows an exponential decay:

$$\begin{aligned} Q(t)=Q_{0}exp(-\frac{t}{R_{eq}C_{eq}})\#(S5) \end{aligned}$$

From Eq. (S1), the current can be expressed as

$$\begin{aligned} I\left( t \right)=-\frac{Q(t)}{R_{eq}C_{eq}}=-\frac{Q_{0}}{R_{eq}C_{eq}}exp(-\frac{t}{R_{eq}C_{eq}})\#(S6) \end{aligned}$$

Thus, the maximum magnitude of the discharge current occurs at t = 0:

$$\begin{aligned} I_{max}=\left| I\left( 0 \right) \right|=\frac{Q_{0}}{R_{eq}C_{eq}}\#(S7) \end{aligned}$$

Because the measured output voltage corresponds to the voltage drop across the load resistor R_Load_,

$$\begin{aligned} V\left( t \right)=I\left( t \right)R_{Load}\#(S8) \end{aligned}$$

Accordingly, the peak output voltage (at t = 0) is

$$\begin{aligned} V_{max}=I_{max}R_{Load}=\frac{Q_{0}}{C_{eq}}\frac{R_{Load}}{R_{eq}}\#(S9) \end{aligned}$$

Finally, substituting Q_0_ = σA_cuticle_ and R_eq_ = R_water_ + R_Load_ (and C_eq_ = C_EDL_) yields

$$\begin{aligned} V_{max}=\frac{{\sigma A}_{cuticle}}{C_{EDL}}\frac{R_{Load}}{R_{eq}+R_{Load}}\#(S10) \end{aligned}$$

This expression matches Eq. (2) in the main text and exhibits that the peak output voltage scales linearly with the effective droplet–cuticle contact area while being modulated by the resistive division between R_water_ and R_Load_.

| **Ref.** | **Surface structure** | **Active zone distance from electrode** | **Dielectric material** | **Electrode material** | **Working principle** | **Output** |
| --- | --- | --- | --- | --- | --- | --- |
| **This work** | Curvilinear channel | Extended to 8 cm | OTS-C_30_ coated PDMS | Hydrogel | Single top electrode | 169 V_PP_ / 125 μA_PP_ |
| W. Xu et al.^[1]^ | Flat surface | Localized | PTFE | Al / ITO | Top-bottom electrodes | 144 V_PP_ / 270 μA_PP_ |
| N. Zhang et al.^[2]^ |  | Localized within 1.8 cm | PTFE | Al | Single top electrode | 62 V_PP_ / 87 μA_PP_ |
| X. Xu et al.^[3]^ |  | Localized | FEP / PET | ITO | Top-bottom electrodes (grid) | 267 V_PP_ / 274 μA_PP_ |
| J. Meng et al.^[4]^ |  | Localized | PTFE | Ti | Single top electrode | 298 V_PP_ / 3.51 mA_PP_ |
| S. Jang et al.^[5]^ |  | Localized | PDMS | Hydrogel | Top-bottom electrodes | 45 V_PP_ / 15 μA_PP_ |
| D. Nguyen et al.^[6]^ |  | Localized | FEP | Al / Carbon | Top-bottom electrodes | 450 V_PP_ / 1.0 mA_PP_ |
| Y. Li et al.^[7]^ |  | Localized within 1.4 cm | PTFE | Water | Top-bottom electrodes (split) | 87 V_PP_ / 90 μA_PP_ |

**Table S1.** Comparison of representative droplet electricity generators (DEGs) reported in the literature and this work. Device structures, working principles, dielectric and electrode materials, effective energy collection zone distances from the electrode, and electrical outputs are summarized.

| **Material** | **δ_d_ [MPa^1/2^]** | **δ_p_ [MPa^1/2^]** | **δ_h_ [MPa^1/2^]** | **Reference** |
| --- | --- | --- | --- | --- |
| Squalane | 16.2 | 0 | 0 | ^[8]^ |
| OTS (C_18_ chain) | 16.4 | 0 | 0 | ^[9]^ |
| PDMS | 15.9 | 0.1 | 4.1 | ^[10]^ |

**Table S2.** Hansen solubility parameters of each material used in artificial cuticle. Each parameter of squalane (C_30_H_62_) was achieved by method of Hoftyzer and Van Krevelen.

| **Alkane** | **Formula** | **Melting point [°C]** |
| --- | --- | --- |
| Dodecane | C_12_H_26_ | −10 |
| Hexadecane | C_16_H_34_ | 18 |
| Octadecane | C_18_H_38_ | 28 |
| Eicosane | C_20_H_42_ | 37 |
| Squalane | C_30_H_62_ | −38 |

**Table S3.** List of alkanes employed in this study with their chemical formulas and melting points.^[11]^ Squalane, which is branched isomer of triacontane (C_30_H_62_), exhibits a significantly lower melting point due to its molecular structure. The phase state (liquid or solid) of each alkane at room temperature (≈ 25°C) is determined by these melting points.

**
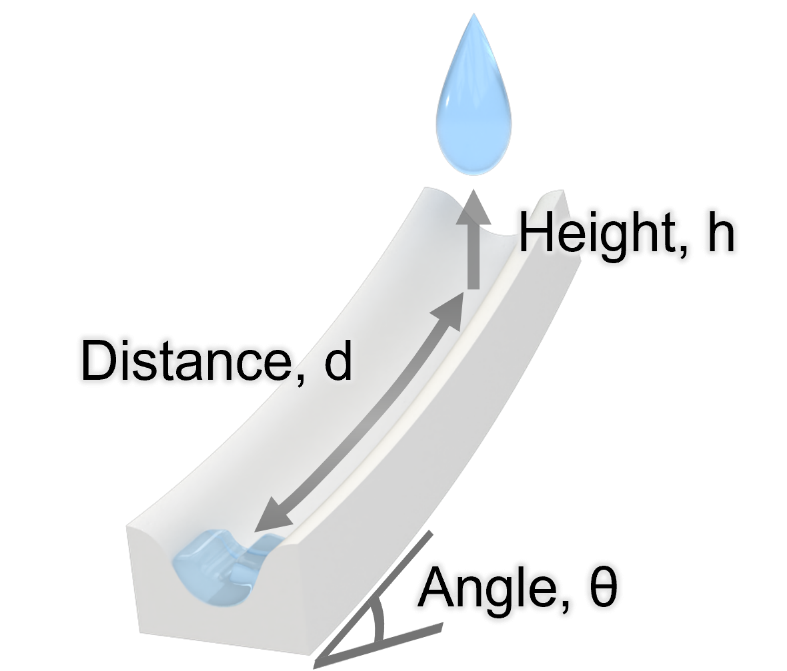
**

**Figure S1.** Experimental parameters of A-DEG, including height (h), distance (d), angle (θ). The d between the droplet impact position and the hydrogel switch electrode was varied from 2 to 8 cm (2, 4, 6, and 8 cm), the h was controlled from 20 to 50 cm (20, 30, 40, and 50 cm), and the θ of the droplet-guiding surface was adjusted from 15° to 60° (15°, 30°, 45°, and 60°).


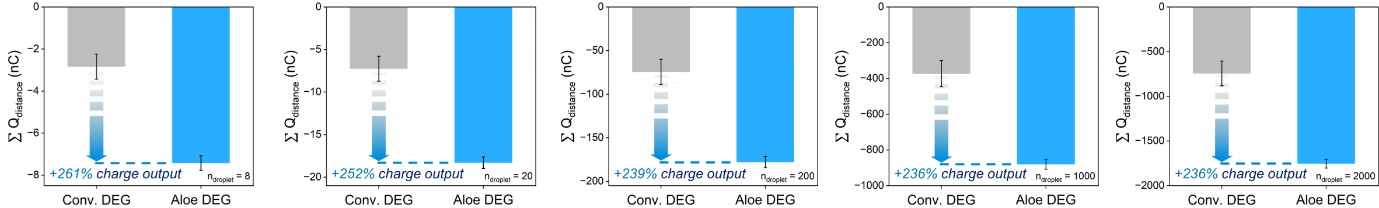


**Figure S2.** Cumulative charge output comparison between conventional and Aloe DEGs with varying droplet impact numbers. The bar graphs illustrate the total transferred charge for the conventional DEG (grey) and Aloe DEG (blue) across different droplet numbers (n_droplet_ = 8, 20, 200, 1000, and 2000). Each bar represents the sum of cumulative charges measured at distances of 2, 4, 6, and 8 cm from the electrode. Despite the exponential increase in the number of droplet samples, the A-DEG consistently outperforms the conventional DEG, maintaining a significant enhancement in charge output ranging from 236% to 261%.


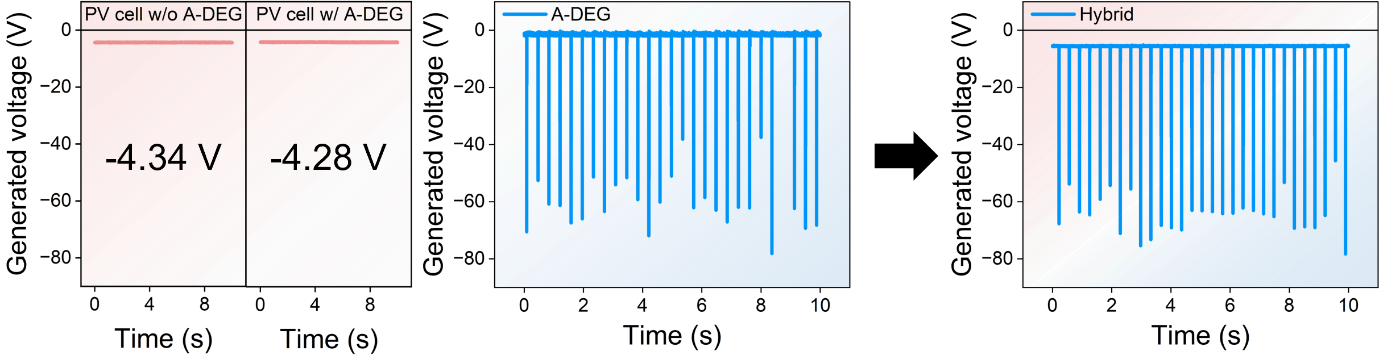


**Figure S3.** Comparison of the generated voltage of a photovoltaic (PV) cell measured under 1000 lx illumination without and with integration of the A-DEG. The PV cell exhibits an average voltage of −4.34 V without the A-DEG and −4.28 V with the A-DEG, indicating a negligible difference and confirming that the optical transparency of the artificial cuticle does not degrade photovoltaic performance. Furthermore, Integration of A-DEG and PV cell demonstrates versatility across diverse weather conditions.


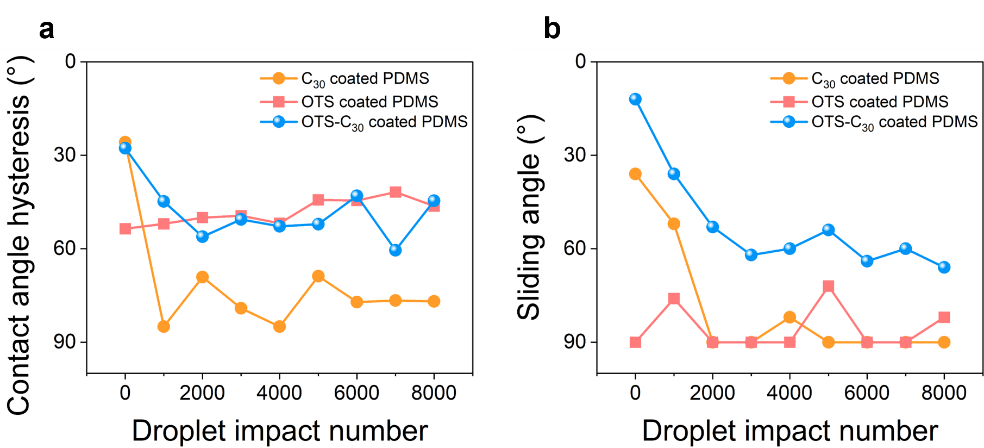


**Figure S4.** Contact angle hysteresis (CAH) and sliding angle for various surface modifications as continuously droplet impact. In here, OTS-C_30_ coating surface modification is used in artificial cuticle, utilized as A-DEG surface. a) OTS and OTS-C_30_ coated PDMS exhibit similar CAH (≈ 45°), while C_30_ coated PDMS shows high CAH exceeding 70°. Following removal of bulk oil layer from OTS-C_30_ coated PDMS surface, CAH trend aligns with behavior of OTS coated PDMS. b) Variation in sliding angle demonstrating superior durability of OTS-C_30_ coating in maintaining surface hydrophobicity against continuous droplet impacts.


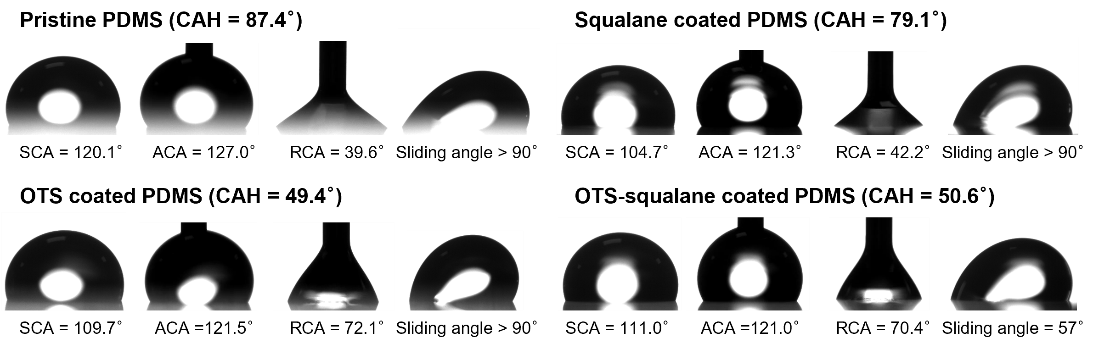


**Figure S5.** Static (SCA), advancing (ACA), receding contact angles (RCA), and sliding angles of various coated surfaces after 3,000 consecutive droplet impacts to remove bulk squalane surface. SCA, ACA, and RCA were measured with 10 μL water droplets on flat surfaces. Sliding angles were measured with 20 μL water droplets on flat surface. Enhanced contact angle hysteresis (CAH), and sliding characteristic was found at OTS-squalane coated PDMS (artificial cuticle).


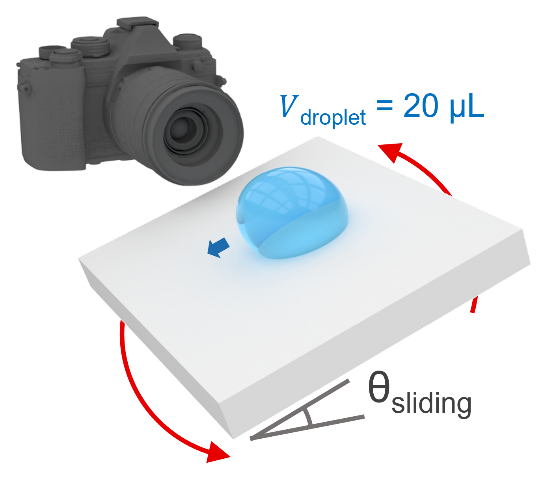


**Figure S6.** Schematic illustration of the sliding angle measurement. A water droplet (𝑉_droplet_ = 20 μL) is placed on the substate, and the surface is gradually tilted. The sliding angle (θ_sliding_) is defined as the angle where the droplet begins to slide downward.


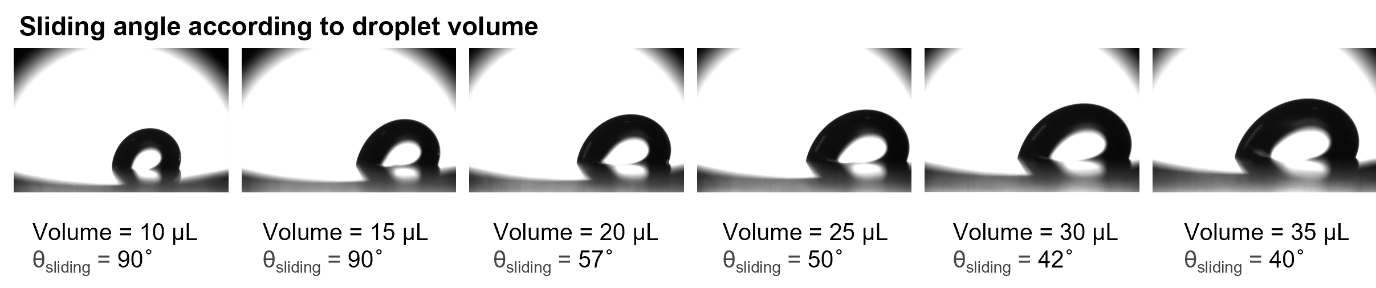


**Figure S7.** Sliding angles (θ_sliding_) of water droplets with diverse volumes on artificial cuticle after 3,000 consecutive droplet impacts.


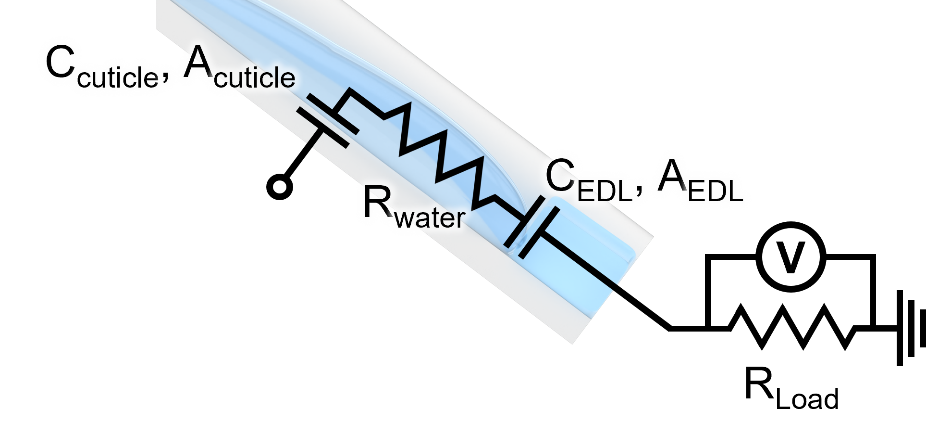


**Figure S8.** Equivalent circuit for A-DEG when switched on. C_cuticle_ and C_EDL_ are the interfacial capacitances formed at the water droplet–artificial cuticle and water droplet–hydrogel electrode interfaces, respectively. Both determined by their effective contact areas (A_cuticle_, A_EDL_​). R_water_​ is the effective electrical resistance of the water droplet. R_Load_​ is the external load resistance, which is set to a near-zero value for current measurements and to 40 MΩ for voltage measurements.


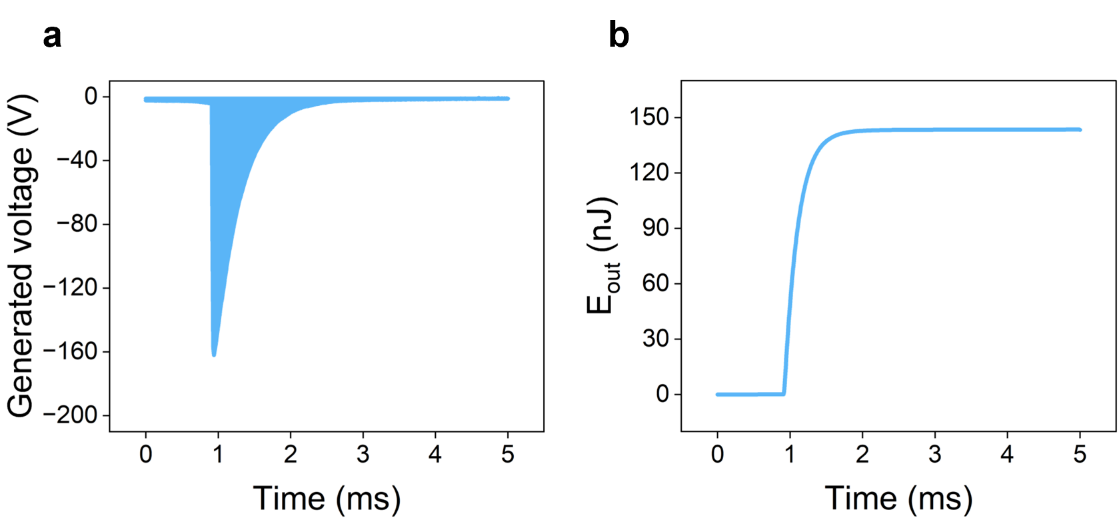


**Figure S9.** (a) A maximum peak voltage of -162 V generated by a single droplet impinging on A-DEG with a resistance of 40 MΩ. (b) An electrical energy output of 138 nJ generated from a single droplet impinging on A-DEG.


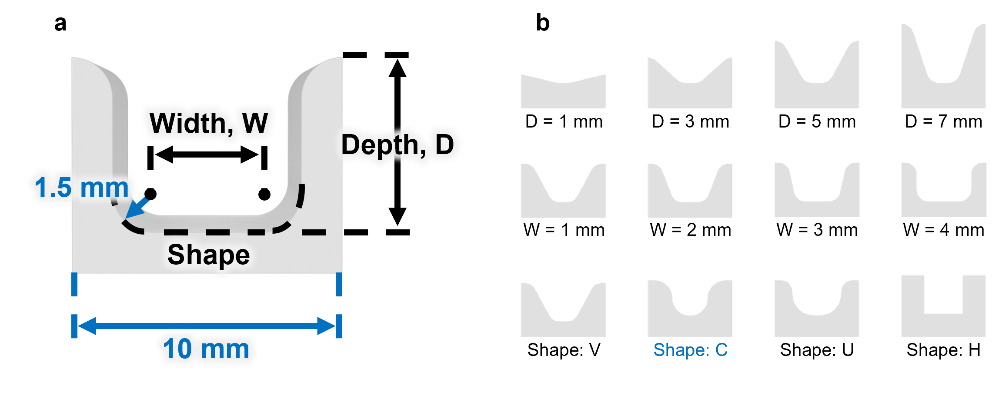


**Figure S10.** Definition of geometric parameters for constructing the artificial midrib, considering width (W), depth (D), and shape. (a) Blue-labeled dimensions fixed as constants (10 mm base width and 1.5 mm fillet radius). Width varied at 1, 2, 3, and 4 mm. Depth varied at 1, 3, 5, and 7 mm. Shape categorized into alphabet-like profiles (V, C, U, H) generated independently of W and D. (b) Front-view schematics illustrating all geometric surfaces. Among all geometries, the C-type channel (blue) exhibiting the most stable droplet sliding and ~130 V output was selected as the optimal design.


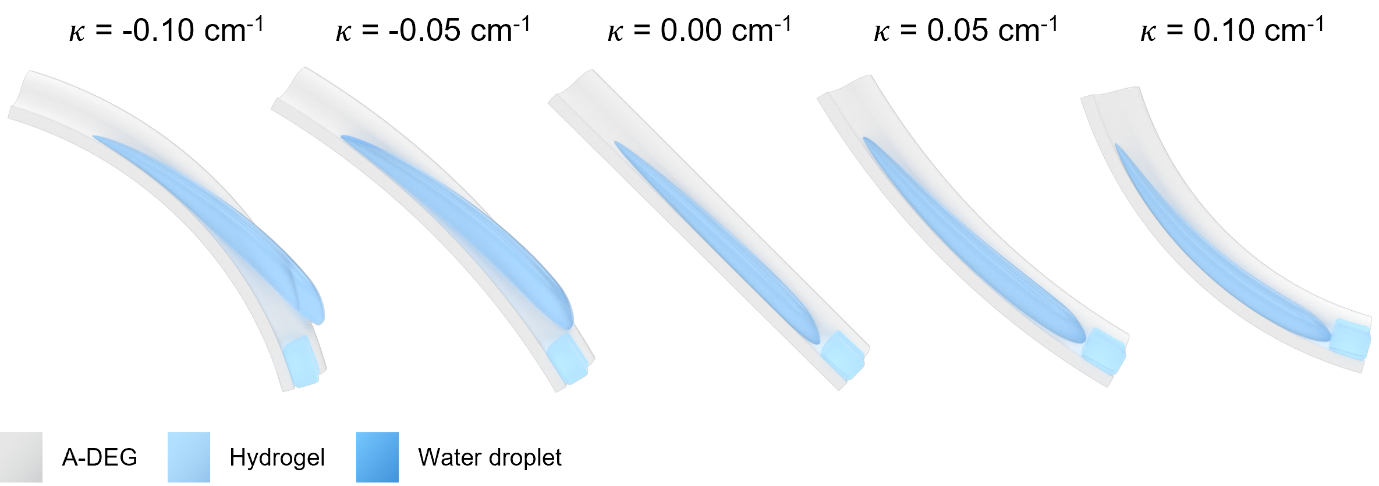


**Figure S11.** Schematic illustration of the 10 cm long A-DEG under various curvature (𝜅). As the curvature becomes negative (convex), the schematic indicates an increased probability of the water losing contact with the electrode compared to the flat or positive curvature states.


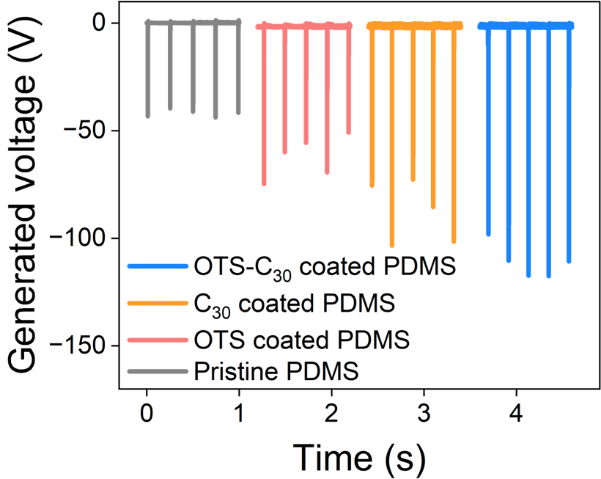


**Figure S12.** Comparison of PDMS-based A-DEGs with pristine, OTS, C_30_, and OTS-C_30_ coatings in terms of voltage generation.


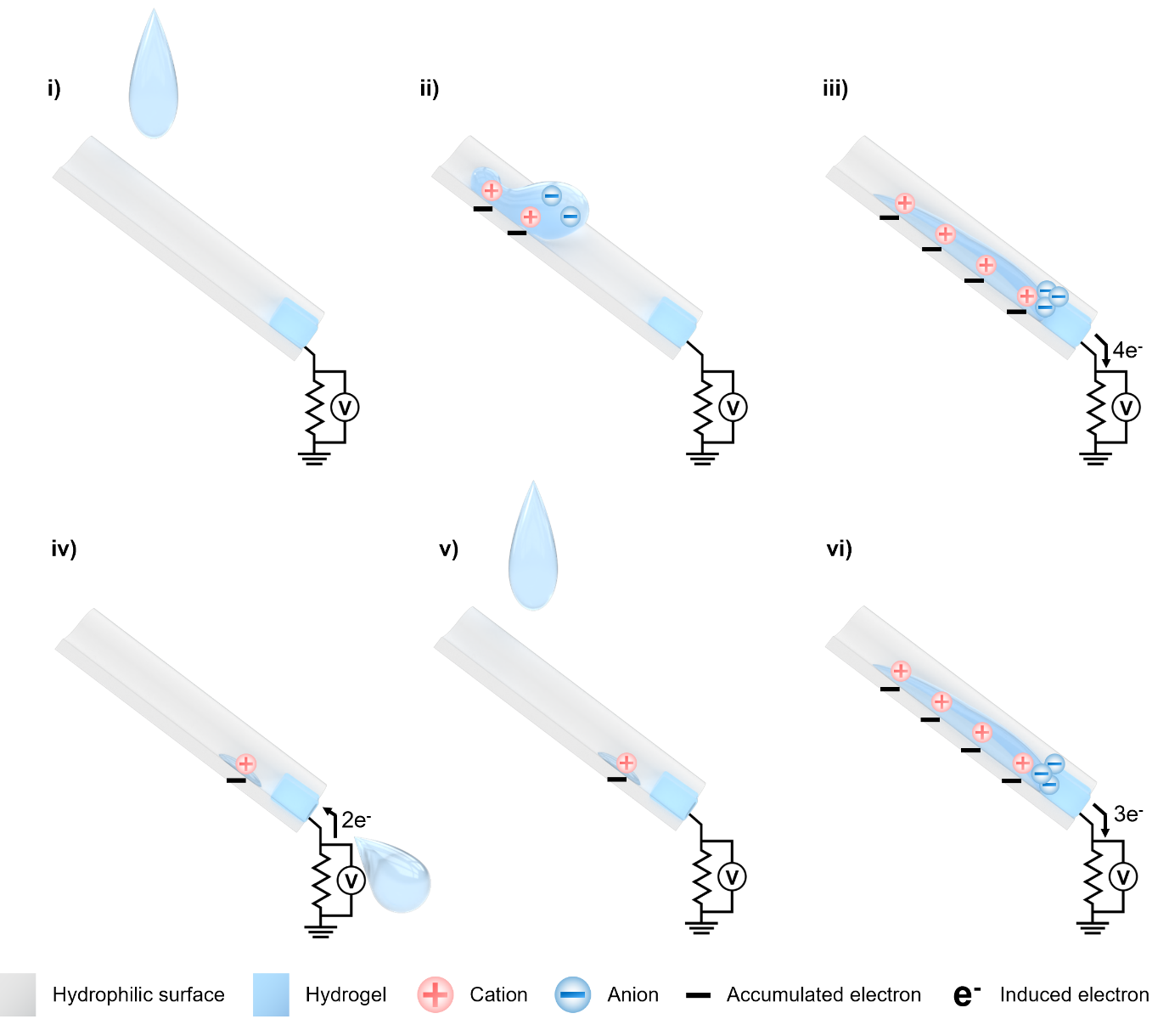


**Figure S13** Energy harvesting behavior of droplet electricity generator (DEG) on hydrophilic surface. (i) Initial state of falling droplet. ii) Contact electrification between droplet and surface. iii) Induced electric charge (4e⁻) via bulk effect. iv) Small amount of pinned water on hydrophilic surface even though droplet slides off. v) Subsequent falling droplet on surface with residual water. vi) Deteriorated charge induction (3e⁻) due to small amount of residual water.


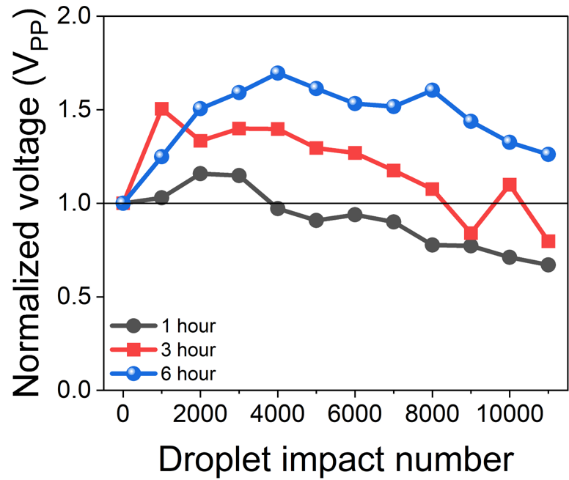


**Figure S14.** Voltage durability as a function of droplet impact number for A-DEGs with different squalane diffusion times (1, 3, and 6 h). The initial voltage increase up to ~3,000 impacts is attributed to the removal of squalane covering the hydrogel electrode and subsequent impact-induced surface charging. Longer diffusion times lead to larger voltage enhancement relative to the initial state and improved voltage retention. After 11,000 droplet impacts, A-DEGs with 1 h and 3 h diffusion times retain ~67% and ~80% of their initial voltages, respectively, whereas the 6 h sample exhibits an increased voltage output of ~126%, demonstrating enhanced long-term durability with extended squalane diffusion.


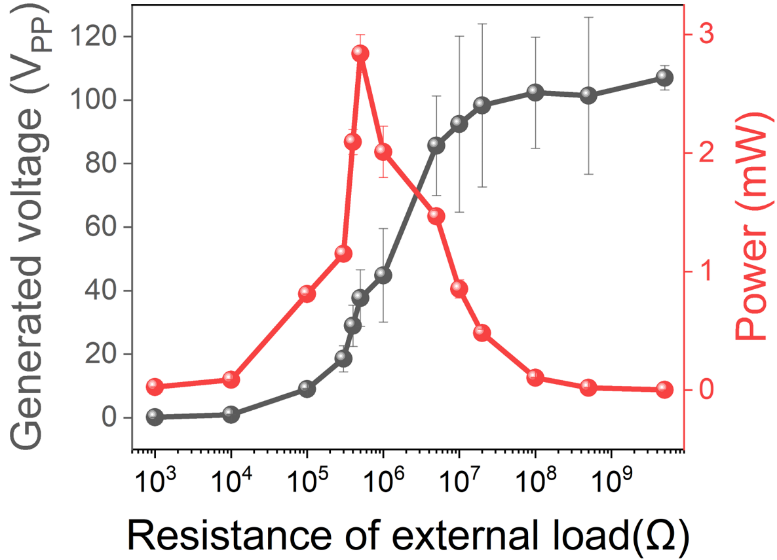


**Figure S15.** A maximum peak power of 2.8 mW of A-DEG at a matched impedance of 500 kΩ, demonstrating its suitability for driving low-power electronics.


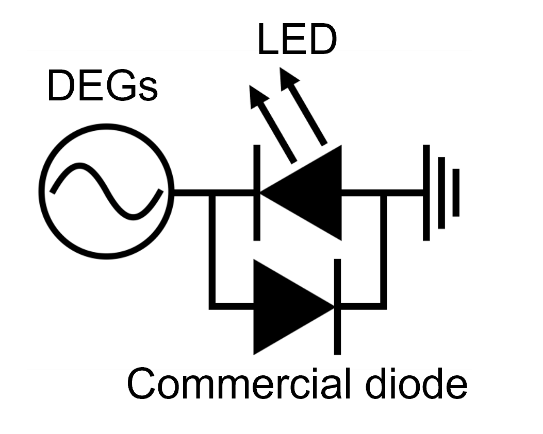


**Figure S16.** Circuit diagram used for the LED illumination test of the DEGs. A commercial diode was inserted to provide a current path during the reverse cycle, thereby enabling smooth charge transport and maximizing LED emission.


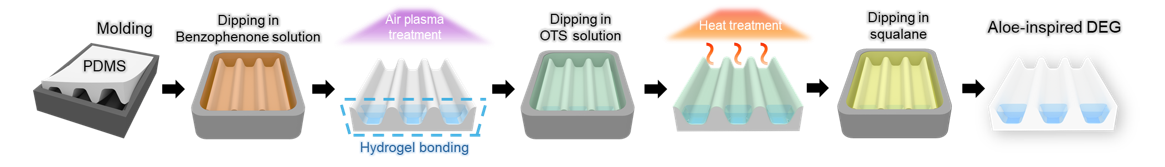


**Figure S17.** Schematic illustration of the A-DEG fabrication process. The pre-molded PDMS substrate is immersed in a benzophenone solution to anchor the hydrogel electrode. Subsequently, the device undergoes plasma treatment to generate hydroxyl groups on the surface. The hydroxy-terminated PDMS is then immersed in an OTS solution, followed by heat treatment to form a functionalized silane layer. Finally, the A-DEG is completed by infusing the surface with squalane oil.

**References**

[1] W. Xu, H. Zheng, Y. Liu, X. Zhou, C. Zhang, Y. Song, X. Deng, M. Leung, Z. Yang, R. X. Xu, *Nature* **2020**, 578, 392.

[2] N. Zhang, H. Gu, K. Lu, S. Ye, W. Xu, H. Zheng, Y. Song, C. Liu, J. Jiao, Z. Wang, *Nano Energy* **2021**, 82, 105735.

[3] X. Xu, P. Li, Y. Ding, W. Xu, S. Liu, Z. Zhang, Z. Wang, Z. Yang, *Energy Environ. Sci.* **2022**, 15, 2916.

[4] J. Meng, L. Zhang, H. Liu, W. Sun, W. Wang, H. Wang, D. Yang, M. Feng, Y. Feng, D. Wang, *Adv. Energy Mater.* **2024**, 14, 2303298.

[5] S. Jang, S. Lee, S. A. Shah, S. Cho, Y. Ra, G. Lee, Y. Lee, D. Choi, *Adv. Funct. Mater.* **2025**, 35, 2411350.

[6] D. C. Nguyen, M. C. Nguyen, D. T. Pham, Z. Ding, S. Na, H. Kim, K. Choi, D. Choi, *Nano Energy* **2025**, 134, 110560.

[7] Y. Li, Y. Zhang, J. Zhang, X. Li, J. Liu, Z. Guo, X. Liu, H. Zheng, *ACS Appl. Mater. Interfaces* **2025**, 17, 42205.

[8] D. W. Van Krevelen, K. Te Nijenhuis, *Properties of polymers: their correlation with chemical structure; their numerical estimation and prediction from additive group contributions*, Elsevier, **2009**.

[9] C. M. Hansen, *Hansen solubility parameters: a user's handbook*, CRC press, **2007**.

[10] T. Uragami, I. Sumida, T. Miyata, T. Shiraiwa, H. Tamura, T. Yajima, *Mater. Sci. Appl.* **2011**, 2, 169.

[11] W. M. Haynes, *CRC handbook of chemistry and physics*, CRC press, **2016**.
